# ELK4 exerts opposite roles in cytokine/chemokine production and degranulation in activated mast cells

**DOI:** 10.3389/fimmu.2023.1171380

**Published:** 2023-07-17

**Authors:** Yuji Huang, Zhehui Zhu, Weize Li, Yiqin Ge, Yanning Li, Juan Wang, Xia Peng, Lihui Lin, Jia Li, Chen-Ying Liu, Li Li

**Affiliations:** ^1^ Department of Laboratory Medicine, Shanghai General Hospital, Shanghai JiaoTong University School of Medicine, Shanghai, China; ^2^ Department of Colorectal Surgery, Shanghai Engineering Research Center of Colorectal Cancer Minimally Invasive Technology, Zhongshan Hospital, Fudan University, Shanghai, China; ^3^ Department of Colorectal and Anal Surgery, Xinhua Hospital, Shanghai Jiao Tong University School of Medicine, Shanghai, China

**Keywords:** mast cell, ELK4, cell cycle, degranulation, MITF, SIRT6

## Abstract

The proliferative potential of mast cells after activation for 3-4h was found to be decreased, which suggests that mast cell degranulation and cell proliferation are differentially regulated. ELK4, a member of the ternary complex factor (TCF) subfamily of Ets transcription factors, is one of the downstream effectors of MAPK signaling that is critical for cell proliferation. And Elk4 has been identified to be vital for macrophage activation in response to zymosan and the transcriptional response to 12-O-tetrade canoyl phorbol-13-acetate (TPA) stimulation in fibroblast. However, the effect of ELK4 on the mast cell transcriptional response to FcϵRI and GPCR mediated activation and its potential functional significance in mast cells remain unclear. Here, we showed that ELK4 expression is downregulated in activated mast cells. *Elk4* knockout suppresses cell proliferation and impedes the cell cycle in bone marrow-derived mast cells (BMMCs), which is associated with decreased transcription of cell cycle genes. Additionally, the transcriptional activation of cytokines and chemokines is diminished while mast cell degranulation is enhanced in *Elk4* knockout BMMCs. Mechanistically, ELK4 might positively modulate *Hdc*, *Ccl3* and *Ccl4* transcription by interacting with MITF and negatively regulate the transcription of degranulation-related genes by complexing with SIRT6. Overall, our study identifies a new physiological role of the transcription factor ELK4 in mast cell proliferation and activation.

## Introduction

Anaphylaxis is a serious allergic reaction that occurs rapidly and includes signs and symptoms that involve the skin, gastrointestinal tract, respiratory system, and cardiovascular system (1). Anaphylaxis can be caused by allergies to foods, insect venoms, medications, and other agents (1). The incidence of food-induced anaphylaxis has increased at an alarming rate, especially in children, over the past several decades and still continues to increase (2, 3). Understanding how anaphylactic shock is regulated at the molecular level is an important step in developing effective prevention and treatment methods.

Mast cells are mononuclear protease granule-containing cells that display FcϵRI, the high-affinity receptor for IgE, and MRGPRX2/MRGPRB2, a G protein-coupled receptor on the cell surface. Mast cell activation by IgE/FcϵRI crosslinking induces degranulation, with the release of inflammatory mediators, including histamine, and the secretion of cytokines (4–6). Histamine is a biogenic amine derived from the decarboxylation of the amino acid histidine, a reaction that is catalyzed by the enzyme L-histidine decarboxylase (HDC). In contrast to histamine, cytokines are members of the group of newly generated mediators, meaning that mast cells in general do not contain large amounts of stored cytokines. Thus, FcϵRI-mediated mast cell activation involves the robust transcription and *de novo* synthesis of cytokines and chemokines, which leads to a late-phase inflammatory response (4–6). In addition, mast cells can be activated by MRGPRX2 activation (7, 8). Individual mast cell responses (mast cell degranulation and cytokine production) were are differentially controlled. Stefanie Klemm et al. have found Bcl10 and Malt1 are essential positive mediators of FcϵRI-dependent mast cell activation that segregates NFκB-induced proinflammatory cytokine production from degranulation (9). And also the Spred/Sprouty family proteins have been identified as negative regulators for ERK activation to negatively regulate mast cell proliferation and cytokine production (10). It suggests ERK activation is responsible for mast cell proliferation and cytokine production.

Information regarding the transcriptional analysis of mast cell development and activation is emerging (11–13). The transcription factors MITF, GATA2 and STAT5 are essential for mast cell development, and NFAT, AP-1 and NF-κB orchestrate the transcriptional response in activated mast cells (11–13). Epigenetic regulators are also involved in regulating mast cells, including the methylcytosine dioxygenase TET2, DNA methyltransferase DNMT3a and histone deacetylase SIRT6 (14–16). Ternary complex factors (TCFs), including ELK-1, ELK3/Net, and ELK4/SAP-1, belong to the Ets transcription factor family. Additionally, TCFs are regulated by Ras-ERK signaling and control cell proliferation in response to serum stimulation (17, 18). Although ELK4 has been implicated in proliferation and cancer (19) and has engaged in the transcriptional response to 12-O-tetradecanoyl phorbol-13-acetate (TPA)-induced fibroblast cell activation and zymosan-induced macrophage activation (20, 21), the extent to which the immediate-early transcriptional response to FcϵRI and GPCR mediated activation in mast cells is ELK4-dependent and the target genes involved have not been systematically investigated.

By analyzing the gene profiles of activated human mast cells, we noticed that *ELK4*, compared with *ELK1* and *ELK3*, is highly expressed in mast cells and that the expression of *ELK4* is significantly downregulated in activated mast cells. These observations encouraged us to explore the regulation and function of ELK4 in mast cells. In this study, we report that ELK4 promotes mast cell proliferation *in vitro*. *Elk4* deficiency in BMMCs impairs gene transcription of inflammatory mediators and cell cycle components but enhances mast cell degranulation. ELK4 interacts with MITF and SIRT6 and might transcriptionally modulate cytokine/chemokine expression and degranulation by cooperating with MITF and SIRT6, respectively. Thus, our study reveals the important but opposite roles of the transcription factor ELK4 in cytokine/chemokine production and mast cell degranulation.

## Materials and methods

### Mice

Conventional *Elk4* knockout mice on a C57BL/6N genetic background were purchased from Cyagen Bioscience. 6- to 12-week-old animals of both sexes were used, and littermate wild-type (WT) mice were used as controls in all experiments. All studies using these mice were approved by the Institutional Animal Care and Use Committee of Shanghai General Hospital affiliated with Shanghai Jiao Tong University School of Medicine and were performed in compliance with ethical guidelines.

### Publicly available data analysis (GSE107316)

Expression Matrix (GSE107316_fpkms.txt) was downloaded from GEO data base (https://www.ncbi.nlm.nih.gov/geo/download/?acc=GSE107316&format=file&file=GSE107316%5Ffpkms%2Etxt%2Egz). This dataset was generated from human mast cells induced from peripheral blood-derived CD34^+^ cells. The human mast cells were left untreated or sensitized overnight with myeloma IgE (0.5 μg/ml) and treated with anti-IgE (1μg/ml) for 2h before harvesting for RNA extraction and subsequent RNA-seq. FPKM was transformed to TPM firstly and differentially expressed genes were analyzed by using limma. Besides the publicly available expression data, no human mast cells were used in our experiments.

### Generation of BMMCs

Bone marrow cells were obtained by flushing bone marrow from the femurs and tibias of mice. Cells were cultured in BMMC media (RPMI 1640 media containing 2 M L-glutamine, 10% foetal bovine serum, 1 M sodium pyruvate, 0.1 M nonessential amino acids, 100 U/mL penicillin, 100 mg/mL streptomycin, 25 M HEPES, 10 ng/mL IL-3, and 10 ng/mL SCF (PeproTech, Cranbury, NJ)). After 4 to 8 weeks of culture, the BMMCs were stained to confirm surface expression of fluorescein isothiocyanate (FITC)–anti-mCD117 (BioLegend) and red-phycoerythrin–anti-mFcϵRI (BioLegend), and >80% of the cells were found to be double positive.

### Isolation and culture of peritoneal cavity mast cells (PCMCs)

Mouse peritoneum was flushed with cold phosphate-buffered saline (PBS), and peritoneal cells were isolated and cultured in BMMC media (RPMI 1640 media containing 2 M mol L-glutamine, 10% foetal bovine serum, 1 M mol sodium pyruvate, 0.1 M mol nonessential amino acids, 100 U/mL penicillin, 100 mg/mL streptomycin, 25 M mol HEPES, 10 ng/mL IL-3, and 10 ng/mL SCF (PeproTech, Cranbury, NJ)) for 3 weeks as previously described (22). Cell purity was routinely checked by cell-surface staining for FcϵRI and CD117, and >85% of the cells were double positive.

### RT−qPCR analysis

5x10^5^ BMMCs were seeded in 24-well plates and sensitized with anti-DNP-IgE(0.5μg/ml) overnight. On the next day, BMMCs were stimulated for 1 hour at 37°C with 100 ng/mL DNP-HSA antigen. Similarly, 5x10^5^ BMMCs were seeded in 24-well plates and stimulated with Compound 48/80 (5 μg/ml) for 1 hour. Total RNA was extracted by using TRIzol (Invitrogen) after activation. One microgram of the isolated RNA was used for complementary DNA (cDNA) synthesis with PrimeScript™ RT Master Mix (TAKARA). Reverse transcription quantitative polymerase chain reaction (RT−qPCR) was then performed using Hieff^®^ qPCR SYBR Green Master Mix (YEASEN) on a 7500 real-time fluorescence quantitative PCR instrument. Experiments were performed in duplicate for each sample, and the mRNA expression was normalized to hprt mRNA. The primer sequences are listed in **Table S1**.

### Immunoprecipitation and western blotting

Cells were lysed with lysis buffer [170 mM NaCl, 50 mM Tris (pH 8.0), 0.5% (v/v) NP-40, 1% Triton X-100, 1 mM EDTA, 5% glycerol, and protease inhibitors] and precleaned with 20 μl of protein Sepharose (GE Healthcare) for 1 hour at 4°C. Antibodies were added to the precleared lysates and allowed to bind at 4°C overnight. Antibody-antigen complexes were precipitated by the addition of Protein Sepharose slurry for 2 hours at 4°C and then washed with washing buffer [100 nM NaCl, 200 mM Tris (pH 8.0), 0.5% (v/v) NP-40, and protease inhibitors] six times and eluted with 2× SDS sample buffer before being subjected to immunoblotting analysis. The following antibodies were used for endogenous co-IP and WB analysis: SIRT6 (Cell Signaling Technology, 12486), ELK4 (Atlas antibodies, HPA028863), and MITF (Cell Signaling Technology, 97800).

### Passive cutaneous anaphylaxis and passive systemic anaphylaxis

For PCA, mice were intradermally injected with 100 ng (in 20 μl) of anti-dinitrophenyl (DNP)-IgE (SPE-7 clone) diluted in PBS in the right ear and 20 μl of PBS in the left ear. Sixteen hours later, the mice were challenged by intravenous injection of 200 μg of DNP-human serum albumin (HSA) (in 100 μl of PBS). For Evans blue dye extravasation experiments, a PCA reaction was carried out as described above, except that 1% Evans blue dye was also included with DNP-HSA (200 µg in 100 PBS) during intravenous injection. Thirty minutes after challenge with DNP-HSA (containing 1% Evans blue), the mice were euthanized, and whole ear pinnae were collected. The ear pinnae were diced into pieces in an Eppendorf tube and incubated in 200 μl of formamide overnight at 55°C. The samples were then centrifuged at 16,200 × g for 10 min. One hundred microlitres of supernatant was quantified with a plate reader by measuring the absorbance at OD 600 nm (23).

For PSA, Mice were sensitized by intravenous injection with 10 μg of anti-DNP-IgE in 100 μL PBS. The following day, mice were intravenously challenged with 100 μg of DNP-HSA in 100 μL PBS. Body temperature was measured with a rectal thermometer after challenge in every 10 minutes for 90 minutes.

### Flow cytometry

For cell-surface labeling, cells were washed with cold PBS and incubated with a blocking antibody for 10 min. Fluorochrome-conjugated antibodies were diluted 1:100 in PBS and incubated with cells on ice for 20 min.

For subcellular labeling, cells were stained using Fix&Perm kit (Multiscence, GAS005/2). 2x10^5^ cells were fixed with 100μL medium A for 15min. After washing with PBS containing 5% FBS, cells were permeabilized with 100μL medium B and stained with IL-6, CCL3, TNFα for 20min respectively. Stained cells were washed with PBS and analyzed on Cytek Aurora instrument.

Antibodies for flow cytometry were as follows: anti-mouse CD45-violetFluor450 (Multi Sciences, AM04512), anti-mouse CD45-PECY7 (BioLegend, 103114), anti-mouse CD19-FITC (Multi Sciences, AM01901), anti-mouse CD3-PE (BioLegend, 100205), anti-mouse CD11b-Percp-cy5.5 (Multi Sciences, AH011B07), anti-mouse GR-1-PE (Multi Sciences, AM0L604), Lamp1-FITC (ProteinTech, FITC-65050), IL-6-eFluor450(ebioscience, 48-7061-82), CCL3-PE(ebioscience, 12-7532-82), TNFα-APC(ebioscience, 17-7321-82).

### β-Hexosaminidase assay

BMMCs and PCMCs were sensitized with anti-DNP-IgE (1 μg/ml) overnight. On the next day, cells were washed with Tyrode’s buffer, seeded in V-shaped 96-well plates, and incubated with DNP-HSA (100 ng/ml) for 1 hour. The supernatant and lysed cell pellet were collected and incubated with p-NAG (4-nitrophenyl N-acetyl-β-D-glucosaminide) substrate for 1.5 hours. The reaction was stopped with 0.2 M glycine (pH 10.7), and absorbance was measured at 405 nm by a spectrophotometer. The percent degranulation was calculated using optical density (OD) values.

### Enzyme-linked immunosorbent assay

The levels of histamine in the culture supernatants were measured using commercial ELISA kits (LDN, BA E-5800R). IL-6 level was detected using ELISA kits (Multi Sciences, 70-EK206/3). TNFα level was detected using ELISA kits (Multi Sciences, 70-EK282/4). CCL3 level was detected using ELISA kits (Multi Sciences, SEA092Mu).

### Histology

Tissues were fixed with 10% formalin and embedded in paraffin. Fixed tissues were cut into 4μm sections, and the paraffin was removed. Sections were stained with haematoxylin-eosin or toluidine blue for light microscopic examinations or mast cell infiltration assays, respectively. The area of the bronchiole wall (Wa) and the perimeter of the bronchiole basement membrane were analyzed by ImageJ (National Institutes of Health). The Wa/Pbm ratio was used to evaluate lung inflammation in asthmatic mice.

The sections were deparaffinized, followed by antigen retrieval with citrate buffer (pH 6.0). Then, 3% hydrogen peroxide and 5% goat serum were used to block endogenous peroxidases and nonspecific antigens, respectively. The primary antibody against MUC5AC (1:500, Abcam, ab3649) was added for incubation overnight at 4°C. The secondary antibody was then applied to the sections for 1 h at room temperature after washing with phosphate-buffered saline (PBS) three times. Finally, the chromogen diaminobenzidine (Beyotime, Haimen, China) was used to detect positive expression of the primary antibody. The sections were ultimately counterstained with haematoxylin and cover slipped. MUC5AC expression in the sections was evaluated and semiquantitatively evaluated by two independent pathologists based on the IHC results. The immunohistochemical staining intensity of MUC5AC was scored as negative (0), weak (1), moderate (2) or strong (3).

### RNA-seq analysis

Total RNA from BMMCs was extracted and used for RNA-seq with a HiSeq 2500 instrument. Differential gene expression analysis was performed with Deseq (comparing resting and IgE-DNP/HSA-induced, wild-type (WT) and *Elk4* KO BMMC samples; adjusted p value (adj-p) <0.05; |Log2 fold change|≥1). The effects of knockout and reconstituted background were estimated by comparing the degree of gene induction under the different conditions and identifying genes that were similarly affected according to the analytical methods in a published paper (20). The basic assumption was that the activity of the genes dependent on a particular transcriptional regulator will be similarly affected by changes in the overactivity or inactivity of that regulator. This method compares the IgE-DNP/HSA-induced fold change in background 1 (*Elk4* WT) (x-axis: fold change in background 1, WT FC) to the difference between the fold change in background 1 and background 2 (y-axis: [fold change in background 1] - [fold-change in background 2]: WT FC- KO FC). Systematic influence of background 2 (*Elk4* KO) will generate a distribution asymmetrically distributed about y=0, and linear regression analysis of the data points above and below y=0 should generate slopes that are significantly nonzero. For perfect dependence of the induction on background 2 (*Elk4* KO), linear regression of the data above y=0 should be r^2 ^= 1. In contrast, differences between the signals of the two backgrounds that arise from random technical variations will be distributed around y=0, and linear regression of the points above or below y=0 should generate a slope of zero. For each comparison, the linear fits of the data points with y>0 (impaired induction in background 2) and y<0 (enhanced induction background 2) were compared to y=0. Data points presenting significant bias were then filtered through an iterative loop where each data point was either included or excluded according to its Euclidean distance to the regression line fit to the data points under investigation.

### Cell proliferation assay

For cell proliferation analysis, WT and Elk4 KO BMMCs were seeded at a density of 6000 cells per well in 96-well plates. The OD value was measured using a microplate reader after the addition of CCK8 (Dojindo, Kumamoto, Japan) for 1h at day 1, day3, day5.

### Cell cycle assay

In preparation for cell cycle distribution analysis, 2× 10^5^ cells were fixed with 70% ethanol and stained with propidium iodide (Beyotime). These cells were then subjected to fluorescence-activated cell sorting using a Cytek Aurora instrument, and data were analyzed using ModFit LT cell cycle analysis software (Verity Software House, Topsham, ME, USA).

### Statistical analysis

Data are expressed as the means ± SD. Statistical comparisons were made by using student’s *t*-test for assays with 2 experimental groups, one-way ANOVA for experiments with over 2 experimental groups, two-way ANOVA for CCK8 assay and Hypergeometrical tests for GO analysis. The ELISA and RT-qPCR assay were performed for 2 or 3 independent experiments. P < 0.05 was considered statistically significant.

## Results

### ELK4 is highly expressed and downregulated in activated mast cells

**Figure 1:**
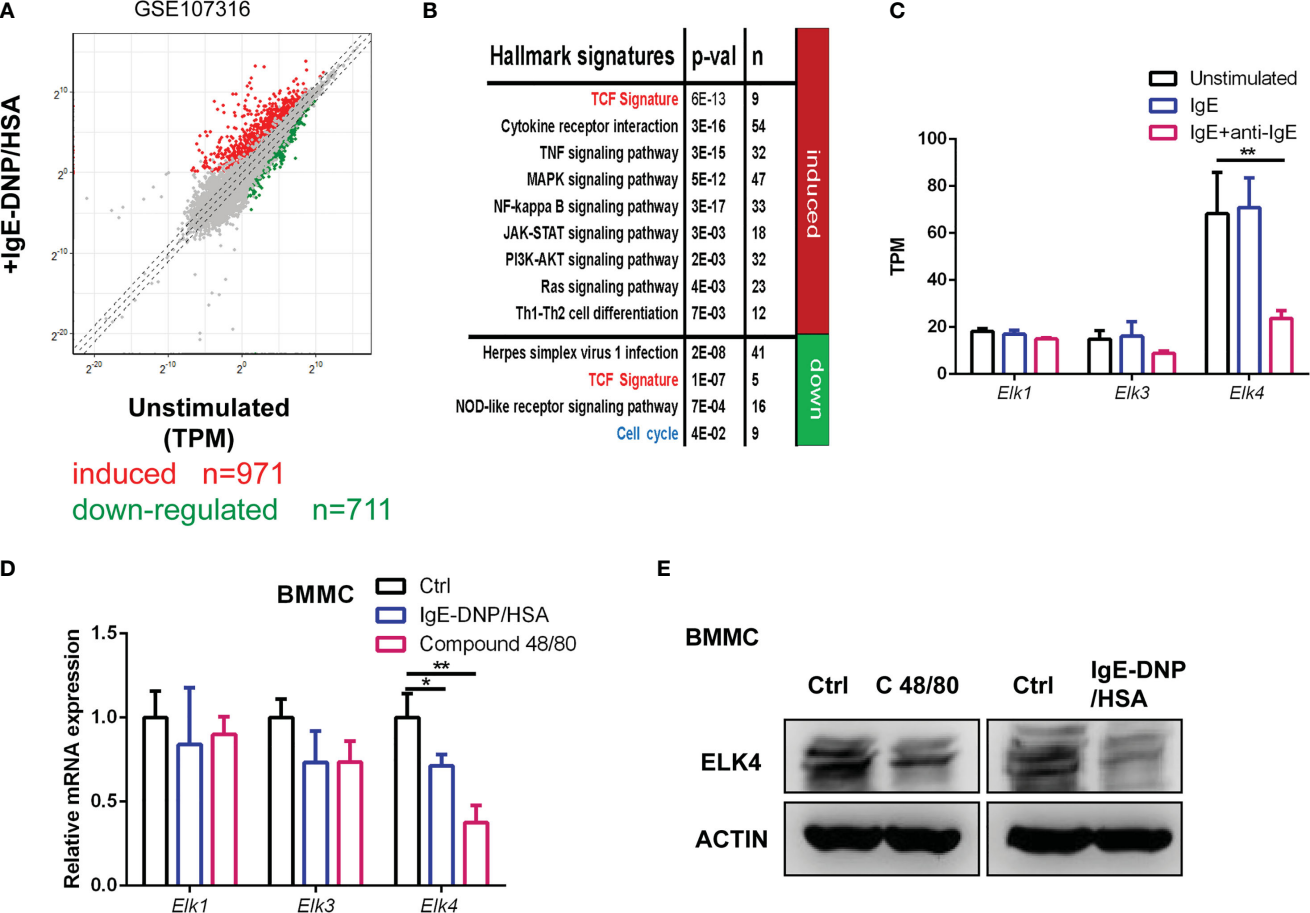
*Elk4* is expressed and downregulated in activated mast cells. **(A)** Scatterplot displaying the induced (red) and downregulated genes (green) after FcϵRI-mediated stimulation in human mast cells (|Log2 fold change|≥1, adj p<0.05). The transcripts per kilobase of exon model per million mapped reads (TPM) were extracted from the GSE107316 dataset. **(B)** Gene ontology analysis of FcϵRI crosslinking-induced and FcϵRI crosslinking-downregulated genes by using the MSigDB hallmark gene set signatures and TCF target gene signature. Hypergeometrical tests were used to assess statistical significance. Bonferroni-adjusted p values are shown. **(C)** The mRNA levels of *ELK1*, *ELK3*, and *ELK4* in unstimulated, IgE-sensitized, and IgE-DNP/HSA-stimulated human mast cells are shown. TPM data were extracted from the GSE107316 dataset. **(D)** qPCR analysis of *Elk1*, *Elk3*, and *Elk4* mRNA expression in unstimulated, IgE-DNP/HSA-stimulated and Compound 48/80-stimulated BMMCs. One-way ANOVA with Dunnett’s multiple comparison test was used to assess statistical significance. Bar, mean; error bar, SD; n = 3; *p<0.05; **p<0.01. **(E)** Western blot analysis of ELK4 protein expression in unstimulated, IgE-DNP/HSA-stimulated and Compound 48/80-stimulated BMMCs.

First, we reanalyzed published gene expression profiles of human mast cells upon FcϵRI-mediated stimulation (GSE107316) (24). A total of 971 genes (fold change≥2, adj-p <0.05) were transcriptionally upregulated, while 711 genes (fold change ≤ 0.5, adj-p <0.05) were downregulated in response to IgE/anti-IgE in stimulated human mast cells (**Figure 1A**; **Table S2A**). The differences in biological processes and KEGG pathways of the differentially expressed genes between unstimulated and stimulated mast cells were obtained by DAVID (https://david.ncifcrf.gov/summary.jsp) enrichment analysis (**Figure 1B**). The IgE/anti-IgE-induced gene set was enriched in gene hallmarks associated with multiple cellular signaling pathways and cytokine receptor interactions (**Figure 1B**; **Table S2B**). In contrast, the downregulated IgE/anti-IgE genes were associated with hallmarks involving the cell cycle and nod-like receptor signaling (**Figure 1B**; **Table S2B**). Consistently, the proliferative potential of mast cell after activation was investigated only for a few hours and was found to be decreased (25, 26). In addition, we used a gene set of TCF target signatures obtained from chromatin immunoprecipitation (ChIP) by TCFs (27) to perform a hypergeometric test (**Table S3**). The IgE/anti-IgE induced and downregulated gene sets were both enriched in genes from the TCF target signature (27) (**Figure 1B**), suggesting that TCFs might be involved in mast cell activation by transcriptionally controlling TCF target genes. By focusing on the TCF transcription factors, we noticed that the expression level of *ELK4* was much higher than the levels of *ELK1* and *ELK3* in unstimulated human mast cells (**Figure 1C**). Furthermore, the public dataset showed that the mRNA level of *ELK4*, but not *ELK1* or *ELK3*, was dramatically downregulated in FcϵRI-activated human mast cells (**Figure 1C**). We further confirmed the downregulation of *Elk4*, but not *Elk1* or *Elk3*, mRNA in activated BMMCs (**Figure 1D**). Western blot analysis showed that the protein level of ELK4 was decreased upon Compound 48/80 and IgE-DNP/HSA stimulation in BMMCs (**Figure 1E**). These data suggest that ELK4 is the main TCF expressed in mast cells and could play a potential role in mast activation and proliferation.

### *Elk4* deficiency leads to cell cycle arrest in BMMCs

**Figure 2:**
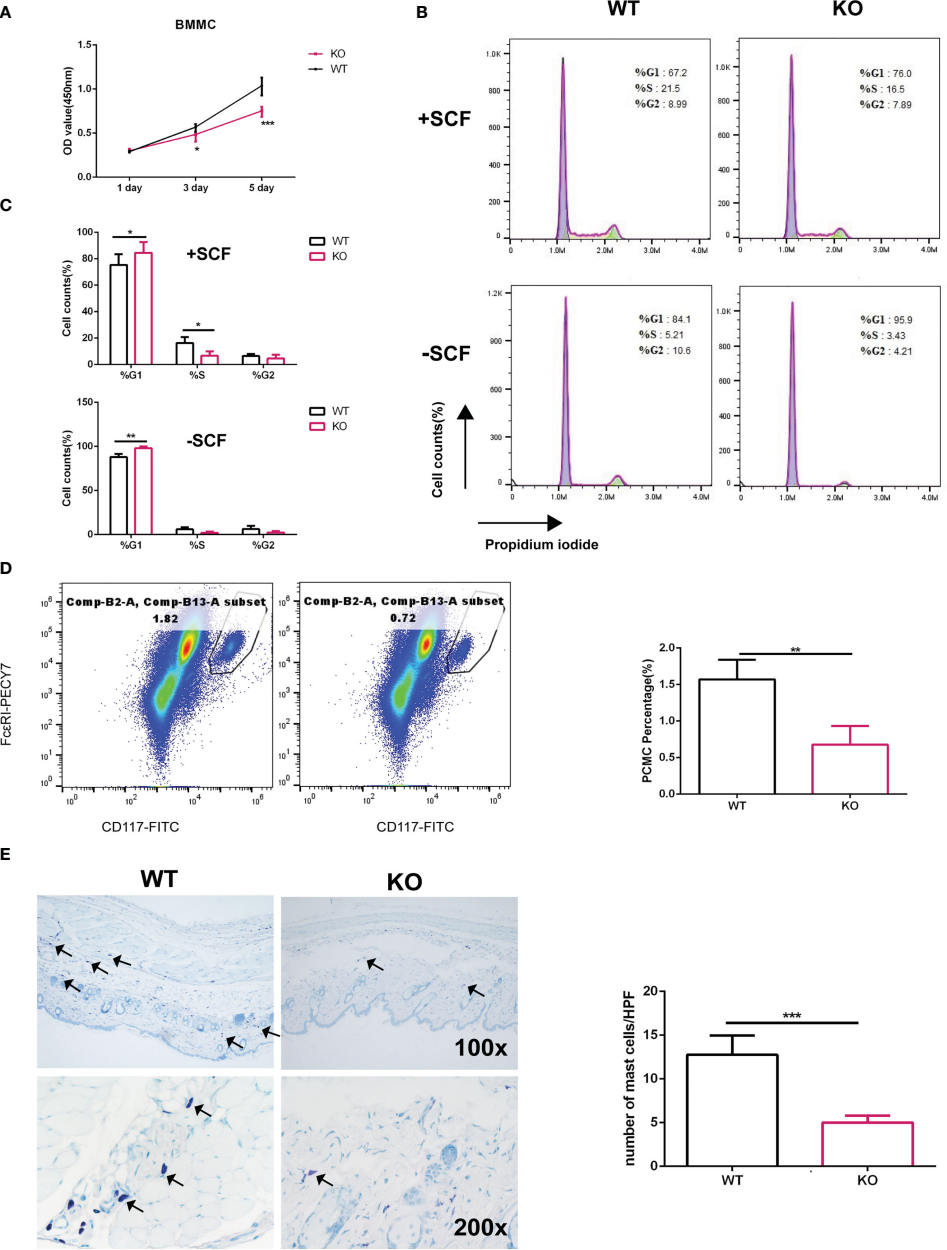
*Elk4* deficiency leads to cell cycle arrest in BMMCs. **(A)** The proliferation of WT BMMCs and *Elk4* KO BMMCs was assessed by CCK8 assay at day 1, day 3 and day 5. Two-way ANOVA with Dunnett’s multiple comparison test was used to assess statistical significance. Bar, mean; error bar, SD; n=3; *p, < 0.05; ***, p<0.001. **(B, C)** Cell cycle analysis of WT BMMCs and *Elk4* KO BMMCs cultured in the presence of IL3 with or without SCF. Flow cytometry data **(B)** and statistical data **(C)** are shown. Bar, mean; error bar, SD; n=3; *p, < 0.05; **p<0.01. **(D)** Percentage of PCMCs in CD45-positive cells from the peritoneal cavities of WT and *Elk4* KO mice. Cells were stained with CD45-eFluor 450, CD117-FITC, and FcϵRI-PECY7 and analyzed by flow cytometry. **(E)** Toluidine blue staining of skin tissues from WT and *Elk4* KO mice. The mast cell number (blue)/high-power field (HPF) was used for statistical analysis. Bar, mean; error bar, SD; n=4; ***p<0.001.

Previously, we reported that ELK4 is critical for epithelial cancer cell proliferation (19). The proliferative potential of mast cell after activation was investigated only for a few hours and was found to be decreased (25, 26). Interestingly, *Elk4* mRNA and ELK4 protein expression both were downregulated after mast cell activation (**Figures 1C–E**). Thus, we explored whether ELK4 could regulate mast cell proliferation. To investigate the potential role of ELK4 in mast cell proliferation and activation, we isolated bone marrow cells from wild-type littermate control (WT), heterozygous (HZ) and homozygous (KO) *Elk4* KO mice that had been induced to differentiate into mast cells in the presence of IL-3 and SCF for 8 weeks (**Supplementary Figure 1A**). Successful induction of BMMCs from WT, HZ and KO mice was confirmed by flow cytometry analysis of CD117 and FcϵRI, as well as by toluidine blue staining (**Supplementary Figures 1B–D**), which suggests BMMCs from both wild-type (WT) and *Elk4* KO mice showed comparable levels of surface CD117 and FcϵRI expression, thus ELK4 does not affect mast cell development (**Supplementary Figures 1B–D**). Deletion of the *Elk4* gene was further confirmed by western blotting analysis of BMMCs from WT, HZ and KO mice (**Supplementary Figure 1E**). Intriguingly, the CCK8 assay showed that *Elk4* KO BMMCs were significantly less proliferative than wild-type BMMCs in normal differentiation culture medium without activation (**Figure 2A**). Cell cycle analysis further showed an increased number of *Elk4* KO BMMCs in G1 phase and a decreased number in S phase compared with wild-type cells (**Figures 2B, C**). SCF affects not only the differentiation but also the proliferation of BMMCs (28). Consistently, G1 arrest of *Elk4* KO BMMCs was also observed in the culture medium without SCF (**Figures 2B, C**).

Next, we counted the number of mast cells in the peritoneal cavity of mice. We isolated PCMCs from the mouse peritoneum and marked the mast cells by staining for CD45, CD117 and FcϵRI. Flow cytometry analysis showed that there were significantly fewer mast cells from the peritoneal cavity (PCMCs) in *Elk4*-deficient mice than in WT mice (**Figure 2D**). Consistent with the analysis of PCMCs, the number of mast cells in the skin of *Elk4*-deficient mice was also reduced compared with that in the skin of WT mice (**Figure 2E**). Notably, we did not observe any effects of *Elk4* deficiency on the numbers of cells of other lineages, such as T/B cells in the spleen and macrophages/neutrophils in the bone marrow (**Supplementary Figure 2**). Altogether, these results suggest that ELK4 promotes mast cell proliferation by regulating the cell cycle.

### *Elk4* deficiency impairs the gene expression of *Hdc, Il6, Tnfα, Ccl3* and *Ccl4* in BMMCs upon FcϵRI-mediated and compound 48/80-induced activation

**Figure 3:**
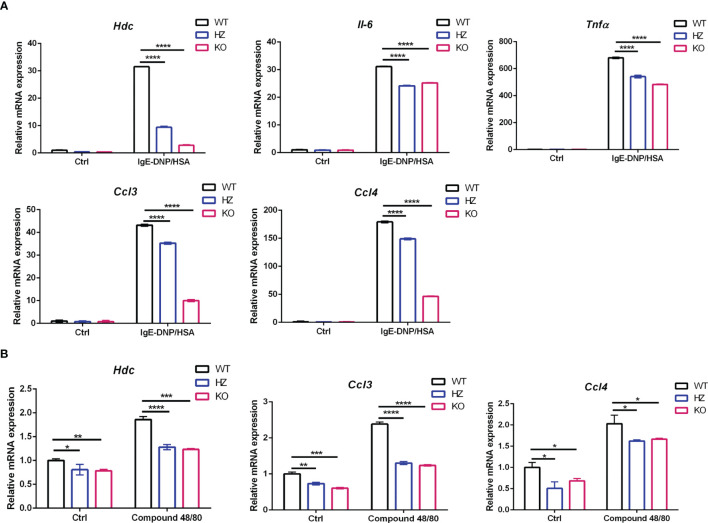
*Hdc*, *Tnfα*, *Il6*, *Ccl3*, and *Ccl4* mRNA expression was impaired in *Elk4* KO BMMCs in response to FcϵRI-mediated and Compound 48/80-induced activation. **(A)** qPCR analysis of *Hdc*, *Il6*, *Tnfα*, *Ccl3*, and *Ccl4* mRNA levels in BMMCs derived from *Elk4* wild-type (WT), heterozygous (HZ), and homozygous (KO) mice in response to FcϵRI-mediated activation. BMMCs were sensitized with anti-DNP-IgE(0.5 μg/ml) overnight and stimulated with DNP-HSA (100 ng/ml) for 1 hour. Bar, mean; error bar, SD; n=3; ****p<0.0001. **(B)** qPCR analysis of the *Hdc*, *Ccl3*, and *Ccl4* mRNA levels in BMMCs derived from *Elk4* WT, HZ, and KO mice in response to Compound 48/80-induced activation. One-way ANOVA with Dunnett’s multiple comparison test was used to assess statistical significance in this figure. BMMCs were stimulated with Compound 48/80 (5 μg/ml) for 1 hour. Bar, mean; error bar, SD; n=3; *p < 0.05; **p<0.01; ***p<0.001.

It has been reported that ELK4 is engaged in the transcriptional response to 12-O-tetradecanoyl phorbol-13-acetate (TPA)-induced activation in fibroblasts and the transcriptional response to zymosan-induced activation in macrophages by regulating *Il10*, *c-fos*, *Egr1* and *Egr3* expression (20, 21). Next, we examined the effect of *Elk4* knockout on the expression of genes related to mast cell activation and function. The *Hdc* gene encodes HDC, the rate-limiting enzyme that is essential for mouse and human histamine synthesis (29, 30). There is only limited knowledge of how Hdc gene expression is regulated. By qPCR analysis, we found that the mRNA level of *Hdc* was decreased in response to IgE-DNP/HSA treatment in *Elk4*-deficient BMMCs (**Figure 3A**). IL-6 is a biomarker of systemic inflammation, metabolic dysfunction, and obesity, and its level is increased in serum and airways in individuals with asthma (31). Tumor necrosis factor α (TNFα), a pleiotropic cytokine that exerts a variety of effects, such as growth promotion, growth inhibition, angiogenesis, cytotoxicity, inflammation, and immunomodulation, has been implicated in various inflammatory conditions (32). Similarly, *Il-6* and *Tnfα* induction were also impaired in response to FcϵRI-mediated activation in BMMCs due to *Elk4* deficiency (**Figure 3A**). CCL3 and CCL4 and their receptors CCR1 and CCR5 play an important role in recruiting other white blood cells, including neutrophils, macrophages (Mφs) and basophils (33). In *Elk4*-deficient BMMCs, we found that *Ccl3* and *Ccl4* induction by FcϵRI-mediated activation was also diminished (**Figure 3A**). Accordingly, IL-6, TNFα and CCL3 protein expression detected by flow cytometry were also impaired in *Elk4* KO BMMCs compared with WT BMMCs (**Supplementary Figure 3A**). Next, we detected IL-6, TNFα and CCL3 protein level by ELISA assay in supernatant of BMMCs induced by IgE-DNP/HSA. Consistently, IL-6, TNFα and CCL3 protein level in supernatant of Elk4-deficient BMMCs stimulated by IgE-DNP/HSA were lower than that in supernatant of WT BMMCs (**Supplementary Figure 3B**). Furthermore, we observed that *Hdc, Ccl3* and *Ccl4* expression was impaired in *Elk4*-deficient BMMCs stimulated by Compound 48/80 (**Figure 3B**). Overall, these data indicate that ELK4 could be indispensable for the transcriptional activation of cytokines and chemokines during mast cell activation.

### *Elk4* deficiency promotes mast cell degranulation and histamine release

**Figure 4:**
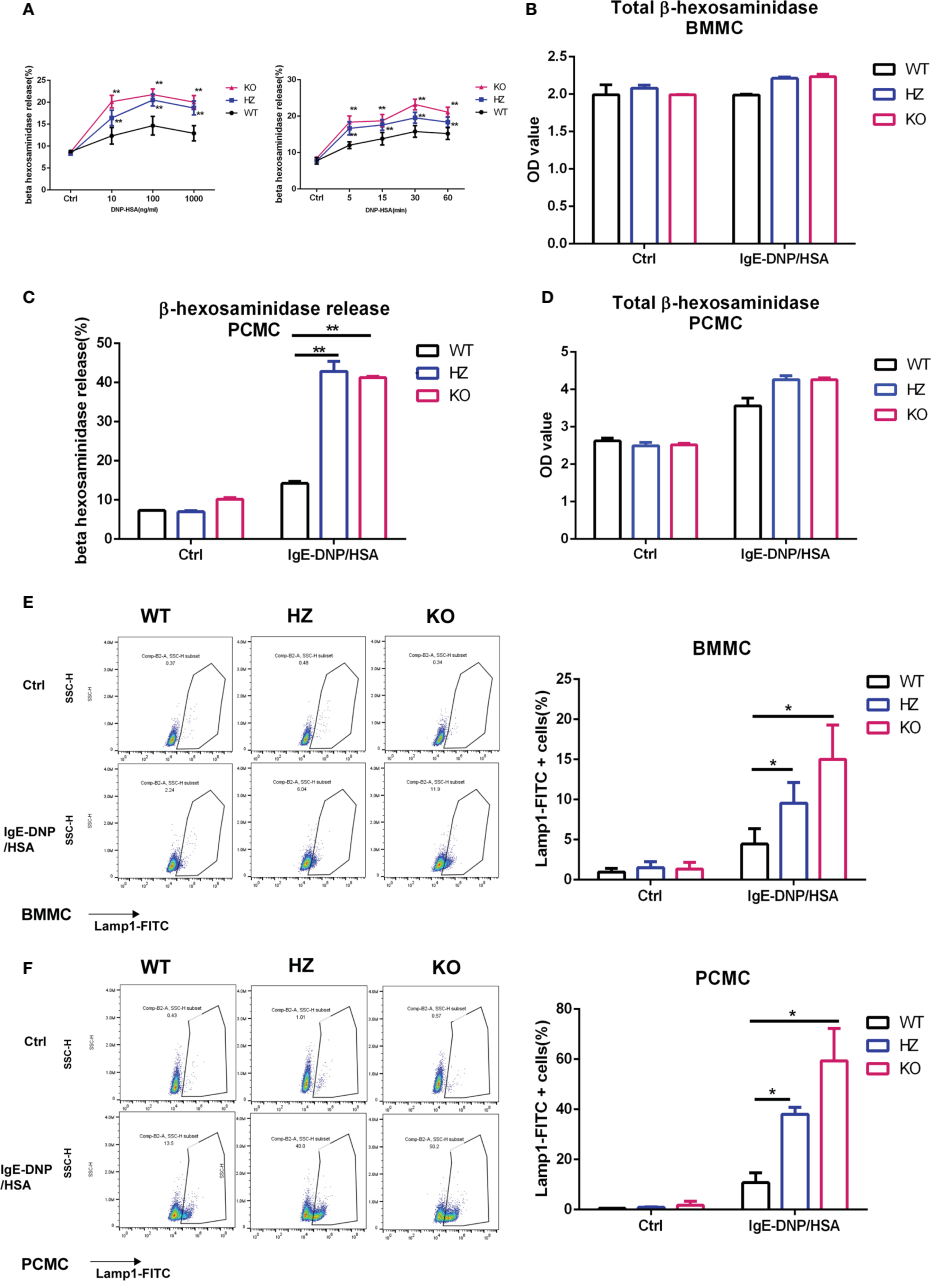
*Elk4* deficiency promotes degranulation and histamine release. **(A)** Time course and dose-dependent curve detection of the beta-hexosaminidase releases. BMMCs were sensitized with anti-DNP-IgE (1 μg/ml) overnight, then stimulated with DNP-HSA (10, 100, 1000 ng/ml) for 60min (Left) or stimulated with DNP-HSA (100 ng/ml) for 5min, 15min, 30min and 60min (Right). Bar, mean; error bar, SD; n=4; **p<0.01. **(B)** The total contents of beta-hexosaminidase in *Elk4* WT, HZ and KO BMMCs are shown. Bar, mean; error bar, SD; n=3; *p < 0.05; **p<0.01. **(C, D)** Degranulation of *Elk4* WT, HZ and KO PCMCs was assessed by beta-hexosaminidase release assay. PCMCs were sensitized with anti-DNP-IgE (1 μg/ml) overnight and stimulated with DNP-HSA (100 ng/ml) for 1 hour. The released contents **(A)** and the total contents **(B)** of beta-hexosaminidase in *Elk4* WT, HZ and KO PCMCs are shown. Bar, mean; error bar, SD; n=2; **p<0.01. **(E, F)** Degranulation of *Elk4* WT, HZ and KO BMMCs **(E)** and PCMCs **(F)** was assessed by cell-surface LAMP1 staining using flow cytometry. Both BMMCs and PCMCs were sensitized with anti-DNP-IgE (1 μg/ml) overnight and stimulated with DNP-HSA (100 ng/ml) for 1 hour before LAMP1 staining. Bar, mean; error bar, SD; n=3; *p<0.05.

Mast cell degranulation leads to the release of inflammatory mediators, including histamine and matrix-degrading proteases. Therefore, we also tested the effect of *Elk4* deficiency on FcϵRI-mediated degranulation. BMMCs were sensitized with anti-DNP IgE overnight and then activated with DNP-HSA. The beta-hexosaminidase release in both *Elk4* WT and KO BMMCs followed the typical bell-shaped dose-response curve when each was treated with increasing concentrations of DNP-HSA for 60 minutes (**Figure 4A**). Release peaked at 30 minutes, followed by a mild decrease at 60 minutes after IgE-DNP/HSA (100ng/mL) treatment (**Figure 4A**). Surprisingly, we observed that the release rate of the β-hexosaminidase enzyme located in BMMCs was significantly elevated in *Elk4* knockout BMMCs upon DNP-HSA stimulation (**Figure 4A**). Avidin-FITC binds to mast cell granules and this conjugated avidin staining technique is a reliable histochemical method for showing mast cell granules (34). We noticed the mildly reduced granularity in unstimulated *Elk4* KO BMMCs compared with WT BMMCs by Avidin-FITC staining during the routine characterization of cultured BMMCs (**Supplementary Figure 1C**). Although there was mildly reduced granularity in un-stimulated *Elk4* KO BMMCs (**Supplementary Figure 1C**), we observed no significant differences in the β-hexosaminidase enzyme between *Elk4* KO and WT BMMCs (**Figure 4B**). Similar results were observed in PCMCs (**Figures 4C, D**). In addition, we examined the expression of cell-surface lysosomal-associated membrane protein 1 (LAMP1/CD107a), which indicates the ability of granules to move to the plasma membrane, in mast cells from *Elk4* KO and WT control mice. Upon DNP-HSA-mediated activation, more *Elk4* KO BMMCs and PCMCs expressed cell-surface LAMP1 than the WT control group, which suggests enhanced mast cell degranulation due to *Elk4* deficiency (**Figures 4E, F**).

Histamine may play various roles in allergic airway inflammation through the receptors H1R, H2R, and H4R in immune cells, including T lymphocytes and dendritic cells. To test whether *Elk4* deficiency affects histamine release upon FcϵRI-mediated activation, we detected histamine by enzyme-linked immunosorbent assay. We found that in *Elk4* KO BMMCs, the rate of histamine release was significantly elevated (**Supplementary Figure 4A**) but the histamine content decreased significantly, which could be due to both the enhanced mast cell degranulation and the downregulation of *Hdc* expression in *Elk4* KO BMMCs (**Supplementary Figure 4B**). Overall, these data suggest that ELK4 could suppress degranulation and histamine release rate in mast cells. Furthermore, degranulation was elevated in *Elk4* KO BMMCs and PCMCs compared to the WT control group in response to Compound 48/80 stimulation (**Supplementary Figures 4C, D**).

### *Elk4* deficiency attenuates anaphylaxis *in vivo*

**Figure 5:**
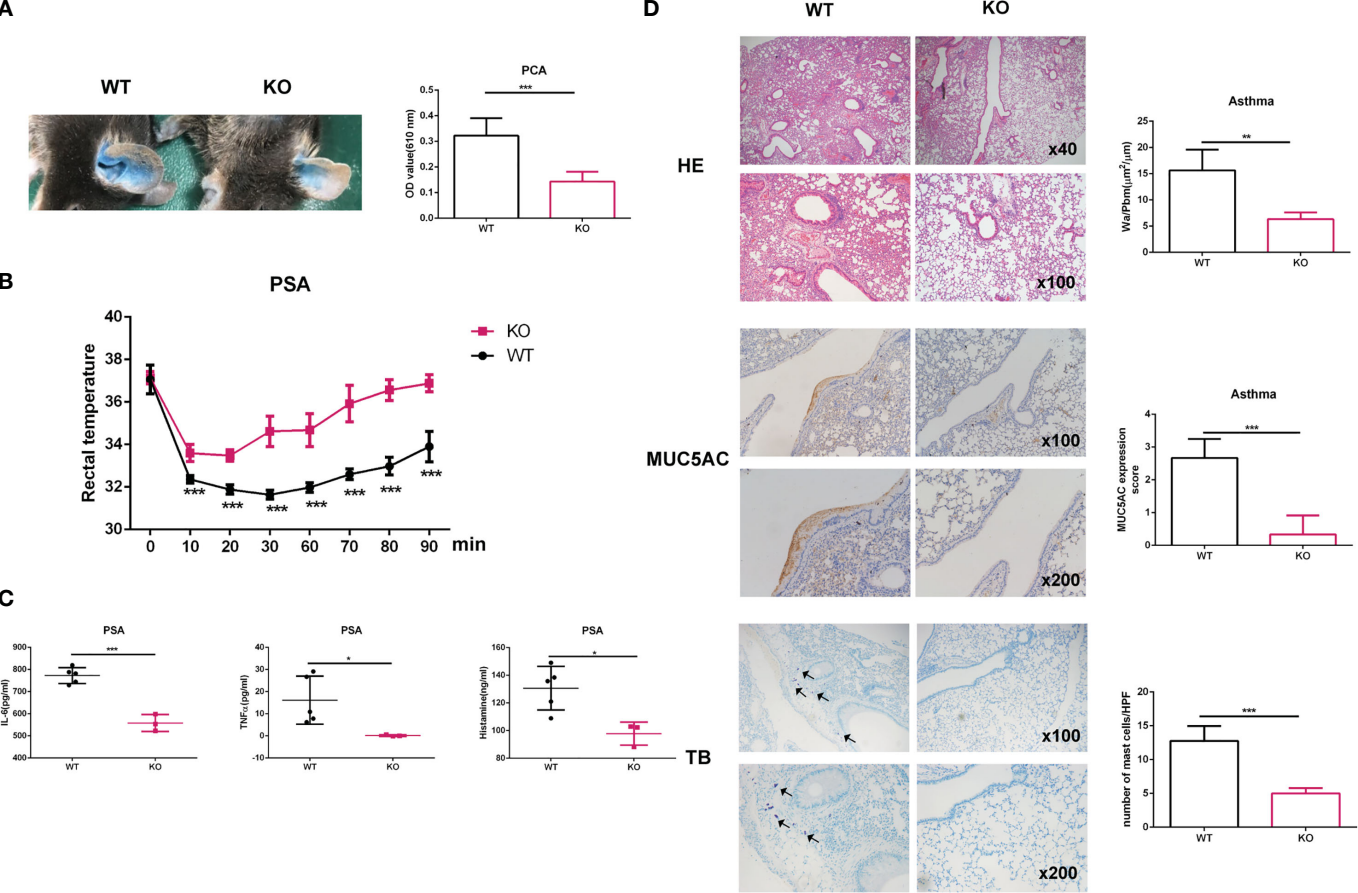
The anaphylactic response in mice was alleviated in *Elk4*-deficient mice. **(A)** Images showing the ear pinnae of *Elk4* WT and KO mice. Evans blue dye extravasation from the ears of *Elk4* WT and KO mice was examined 30 min after intravenous DNP-HSA (containing 1% Evans blue) administration. Evans blue dye extravasation was quantified by measuring the optical density at 610 nm (OD610nm)/weight. Bar, mean; error bar, SD; n=6; ***p<0.001. **(B, C)** WT and *Elk4* KO mice were sensitized with 10 μg anti-DNP-IgE and challenged with 100 μg DNP-HSA. Changes in body temperature and serum levels of IL-6, TNFα and histamine were determined. Bar, mean; error bar, SD; n=3-5; *p<0.05; ***p<0.001. **(D)** HE staining, IHC analysis of MUC5AC and toluidine blue staining of lung sections from *Elk4* WT and KO mice with OVA-induced asthma. The area of the bronchiole wall (Wa) and the perimeter of the bronchiole basement membrane were analyzed by ImageJ. Student’s t test was used to assess statistical significance in the figure. Bar, mean; error bar, SD; n=3; **p<0.01; ***p<0.001.

Next, we studied whether *Elk4* deficiency influences mast cell function during anaphylaxis *in vivo.* We sensitized *Elk4* KO and littermate control mice by intradermally injecting IgE-DNP into their ear pinnae. One day later, we induced mast cell activation by tail vein injection of DNP-HSA and found that the extravasation of Evans blue dye was significantly lower in *Elk4* KO mice than in controls, indicating reduced vascular leakage (**Figure 5A**). We also tested whether *Elk4* deficiency could affect the severity of PSA. We measured rectal temperature as an indicator of PSA. The IgE-sensitized WT mice showed a progressive decrease in rectal temperature within 10 minutes after antigen challenge, while *Elk4* KO mice showed an attenuated response (**Figure 5B**). Accordingly, serum levels of IL-6, TNFα and histamine were higher in WT mice than those in *Elk4* KO mice (**Figure 5C**). Mast cells are one of the key cells in the inflammation of allergic asthma. Elevated serum IgE level is an important feature of allergic asthma. IgE causes chronic airway inflammation mainly through the activation of FcϵRI on effector cells such as mast cells and basophils. We then established a mouse model of allergic asthma induced by exposure to ovalbumin (OVA) as previously described (35). The mice were sensitized and challenged with OVA. Mouse lung sections were examined by haematoxylin and eosin staining, toluidine blue staining, and MUC5AC staining. HE analysis showed that *Elk4* deficiency significantly decreased the thickness of the smooth muscle layer and goblet cell accumulation in the airways compared with those in WT mice (**Figure 5D**, upper panel). Additionally, the area of the bronchiole wall (Wa) and the perimeter of the bronchiole basement membrane were semiquantitatively analyzed, which showed that the Wa/Pbm ratio in the *Elk4*-deficient mice was significantly lower than that in WT mice with asthma (**Figure 5D**, upper panel). Similarly, MUC5AC was expressed at higher levels in WT mice than in *Elk4* KO mice with asthma (**Figure 5D**, middle panel). Consistent with the observations in the skin tissue, the number of mast cells in the lung tissue from *Elk4* KO mice was much lower than that in lung tissue from WT mice with asthma (**Figure 5D**, lower panel). These data suggest that *Elk4* deficiency attenuates anaphylaxis *in vivo*, which could be due to the reduction in both the number of mast cells and cytokine expression in mast cells.

### ELK4 modulates FcϵRI-mediated gene transcription

**Figure 6:**
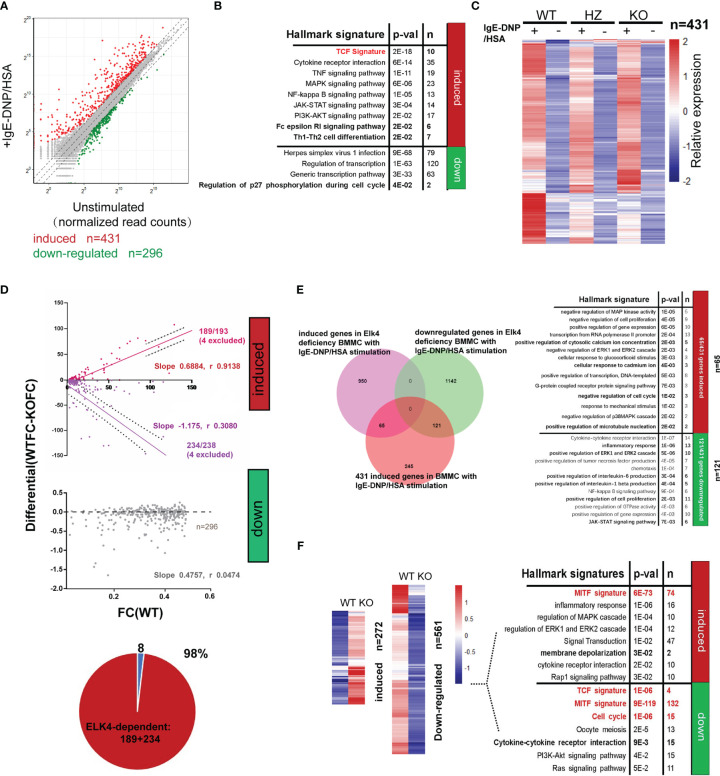
*Elk4* deficiency affects the FcϵRI-mediated transcriptional response in BMMCs. **(A)** Scatterplot displaying the induced (red) and downregulated genes (green) before and after FcϵRI-mediated stimulation in WT BMMCs (|Log2 fold change|≥1, adj p<0.05). **(B)** Gene ontology analysis of FcϵRI crosslinking-induced and FcϵRI crosslinking-downregulated genes in WT BMMCs by using the MSigDB hallmark gene set signatures and TCF target gene signature. Hypergeometrical tests were used to assess statistical significance. Bonferroni-adjusted p values are shown. **(C)** Heatmap representation of the relative mean expression levels of the 431 IgE-DNP/HSA-induced genes in *Elk4* WT and KO BMMCs. **(D)** Identification of ELK4-dependent genes by comparison with the IgE-DNP/HSA induction ratio in *Elk4* WT and KO BMMCs. ELK4-dependent genes (red, purple) exhibited a systematic relationship between their degree of induction in the two contexts, whereas the others (grey) did not. Slope and Spearman r value are indicated. **(E)** Gene ontology analysis of the ELK4-dependent (fold change>1.5 or <0.66, adj p<0.05 in *Elk4* KO BMMCs) FcϵRI-crosslinking induced genes in BMMCs by using the MSigDB hallmark gene set signatures. Hypergeometrical tests were used to assess statistical significance. Bonferroni-adjusted p values are shown. **(F)** Heatmap representation of the relative mean expression levels of the differentially expressed genes between unstimulated *Elk4* WT and KO BMMCs. Gene ontology analysis of the differentially expressed genes was further performed by using the MSigDB hallmark gene set signatures and MITF and TCF target gene signatures. Hypergeometrical tests were used to assess statistical significance. Bonferroni-adjusted p values are shown.

To comprehensively investigate the role of ELK4 in terms of gene expression and function in mast cells, we performed RNA-seq analysis of WT/*Elk4* KO BMMCs and WT/KO BMMCs with FcϵRI-mediated stimulation. A total of 431 genes (fold change≥2, adj-p <0.05) were transcriptionally upregulated, while 296 genes (fold change ≤ 0.5, adj-p <0.05) were downregulated in response to IgE-DNP/HSA in WT BMMCs (**Figure 6A**; **Table S4A**). We observed a similar gene ontology enrichment pattern among the differentially expressed genes between the unstimulated and stimulated BMMCs by DAVID enrichment analysis compared to human mast cells (**Figures 1B**, **6B**). The FcϵRI-mediated induced genes were also enriched in the TCF signature, while those that were downregulated were related to the cell cycle (**Figure 6B**), which was consistent with a previously published dataset (**Figure 1B**; **Table S4B**). Furthermore, RNA-seq analysis revealed that the global transcriptional response to IgE-DNP/HSA was substantially altered in *Elk4* KO BMMCs (**Figure 6C**; **Table S4C**). By using an iterative pipeline to compare the induction of gene expression between the two different backgrounds (20), we partitioned the genes induced by IgE-DNP/HSA in WT BMMCs into the ELK4-dependent and ELK4-independent groups. The basic assumption of this statistical dependence analysis was that the transcription of genes dependent on the same transcription factor should be similarly affected by changes in the activity or abundance of this transcription factor (20). Surprisingly, almost all IgE-DNP/HSA-induced gene expression was dependent on ELK4, whereas FcϵRI-mediated downregulated gene expression showed no obvious ELK4 dependence (**Figure 6D**; **Table S4D**). Notably, *Elk4* deficiency led to extensive variation in the transcriptome in IgE-DNP/HSA-stimulated BMMCs (**Figure 6E**). Over 2100 genes were dysregulated in the IgE-DNP/HSA-stimulated *Elk4* KO BMMCs (fold change ≥1.5 or ≤0.66, adj-p <0.05) (**Figure 6E**). By focusing on the genes induced by IgE-DNP/HSA in BMMCs, 121 genes were downregulated, while 65 were upregulated by *Elk4* deficiency (**Figure 6E**; **Tables S4E, F**). Intriguingly, the 65 upregulated genes were enriched in ‘positive regulation of cytosolic calcium ion concentration’ and ‘positive regulation of microtubule nucleation’, both of which are correlated with enhanced mast cell degranulation (36, 37) (**Figure 6E**). In contrast, the 121 downregulated genes were enriched in ‘inflammatory response’ and ‘chemotaxis’, which was consistent with our previous observation (**Figures 6E**, **3A**).

*Elk4* deficiency also altered the basal expression of various genes in unstimulated BMMCs (**Figure 6F**). A total of 561 genes (fold change ≤ 0.5, adj-p <0.05) were transcriptionally downregulated, while 272 genes (fold change≥2, adj-p <0.05) were upregulated in unstimulated *Elk4* KO BMMCs (**Figure 6F**; **Tables S4G, H**). Consistent with the proliferative defect in *Elk4* KO BMMCs, genes with reduced basal expression in *Elk4* KO BMMCs were enriched in gene hallmarks and terms associated with the cell cycle (**Figure 6F**). qPCR analysis further confirmed the decreased mRNA levels of *Ccna2*, *Ccnb2*, *Cdk2*, *Cdk6* and *E2f2*, which could account for *Elk4* KO BMMC G1 arrest (**Supplementary Figure 5**). Above all, these data demonstrate that ELK4 not only mediates the acute transcriptional response during mast cell activation but also modulates the cell cycle in unstimulated mast cells.

### ELK4 cooperates with MITF and SIRT6 to regulate cytokine/chemokine production and degranulation

**Figure 7:**
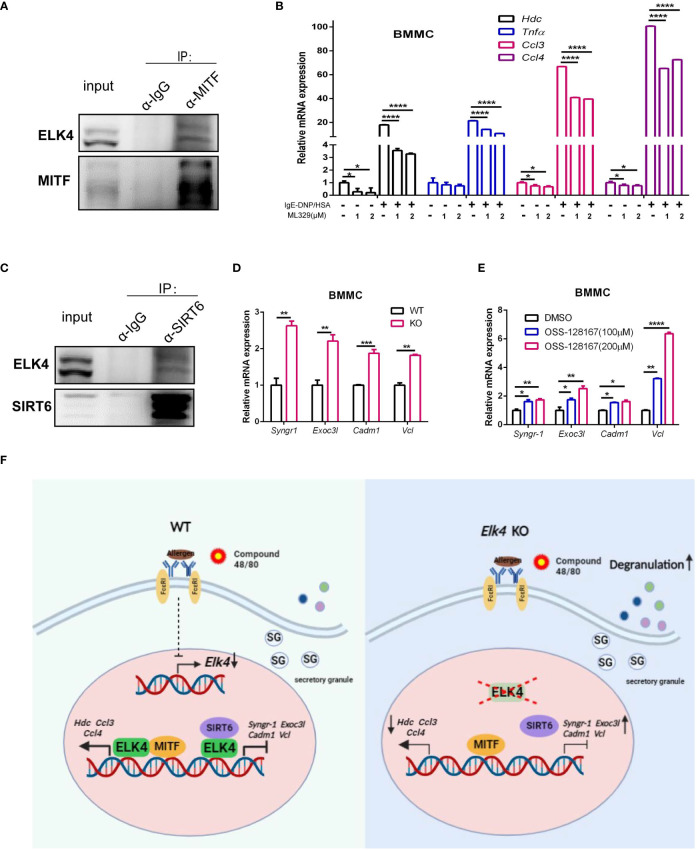
ELK4 interacts and might cooperate with MITF and SIRT6 to regulate mast cell activation and degranulation. **(A)** Coimmunoprecipitation analysis of endogenous MITF and ELK4 proteins in MC/9 mast cells. **(B)** qPCR analysis of the *Hdc*, *Tnfα*, *Ccl3*, and *Ccl4* mRNA levels in BMMCs treated with ML329 in response to FcϵRI-mediated stimulation. One-way ANOVA with Dunnett’s multiple comparison test was used to assess statistical significance. Bar, mean; error bar, SD; n=3; *p < 0.05; ****p<0.0001. **(C)** Coimmunoprecipitation analysis of endogenous SIRT6 and ELK4 proteins in MC/9 mast cells. **(D)** qPCR analysis of *Syngr1*, *Exoc3l1*, *Cadm1*, and *Vcl* mRNA levels in *Elk4*-deficient BMMCs. Student’s t test was used to assess statistical significance. Bar, mean; error bar, SD; n=3; **p<0.01. **(E)** qPCR analysis of *Syngr1*, *Exoc3l1*, *Cadm1*, and *Vcl* mRNA levels in BMMCs treated with OSS-128167. * indicates p<0.05; ** indicates p <0.01;***indicates p<0.001. One-way ANOVA with Dunnett’s multiple comparison test was used to assess statistical significance. Bar, mean; error bar, SD; n=3; *p < 0.05; **p<0.01; ****p<0.0001. **(F)** Schematic diagram depicting the downregulation of *Elk4* expression in activated mast cells and the effect and potential mechanism of ELK4 on mast cell activation.

Our findings that *Elk4* deficiency impairs *Hdc* and cytokine expression in mast cells prompted us to explore whether ELK4 cooperates with key transcription factors known to function in mast cells. One such key TF in mast cells, MITF, has been reported to be required for *Hdc* gene expression and histamine synthesis (38). Upon *Elk4* deficiency, more genes associated with the MITF target signature [assessed by chromatin immunoprecipitation (39)] were enriched in the downregulated gene set in *Elk4*-deficient BMMCs (**Figure 6E**; **Table S5**). Since the protein extraction of the primary BMMCs was relatively low, we used a MC/9 mast cell line for coimmunoprecipitation assay. Through a coimmunoprecipitation assay with ELK4 and MITF, we found that endogenous ELK4 was coimmunoprecipitated by endogenous MITF in MC/9 mast cells, which implied that ELK4 could interact with MITF to cooperatively modulate gene transcription (**Figure 7A**). Consistently, treatment with ML329, a MITF-specific inhibitor, showed a similar suppressive effect on the transcription of *Hdc, TNFα, Ccl3, and Ccl4* in response to FcϵRI-mediated activation of BMMCs (**Figure 7B**). Intriguingly, mast cell degranulation was not affected by ML329 treatment, which indicated that MITF was not involved in the effect of ELK4 on mast cell degranulation (**Supplementary Figure 6A**). We also examined the interaction between ELK4 and other key TFs functioning in mast cells and found that ELK4 could also interact with the histone deacetylase SIRT6 (**Figure 7C**). Recently, SIRT6 was revealed to be a negative regulator of FcϵRI-mediated degranulation in mast cells (14). Thus, we first confirmed the effect of the SIRT6-specific inhibitor OSS_128167 on FcϵRI-mediated mast cell degranulation and found that OSS_128167 treatment significantly exaggerated FcϵRI-mediated mast cell degranulation in BMMCs (**Supplementary Figure 6B**). Furthermore, both OSS_128167 treatment and *Elk4* KO increased the expression of genes associated with degranulation, including *Syngr*-1, *Exoc3l1*, *Cadm1* and *Vcl*, in BMMCs (**Figures 7D, E**). Syngr1, encoding an integral membrane protein, plays a role in regulating vesicle exocytosis, modulates the localization of synaptophysin/SYP into microvesicles, and affects microvesicle formation and maturation (40). EXOC3L1 (exocyst complex component 3 like 1) is a component of the exocyst complex, an evolutionarily conserved multisubunit protein complex that has been implicated in molecular trafficking and tethering secretory vesicles to the plasma membrane (41). Cell adhesion molecule 1 (CADM1), also known as SynCAM1, controls actin cytoskeleton assembly and induces mast cell degranulation and IL-6 secretion (42, 43). VCL, also known as vinculin, can interact with F-actin in both the recruitment of actin filaments to the growing focal adhesions and the capping of actin filaments to regulate actin dynamics (44). Mast cell degranulation is supported by dynamic conversions between actin polymerization and depolymerization (45). Therefore, we speculate that ELK4 might interact with and recruit SIRT6 as an epigenetic corepressor to inhibit the gene transcription of *Syngr1*, *Exoc3l1*, *Cadm1* and *Vcl*, which leads to the suppression of mast cell degranulation. Altogether, these data suggest that ELK4 suppresses mast cell degranulation but also promotes cytokine/chemokine production, probably by cooperating with SIRT6 and MITF, respectively (**Figure 7F**).

## Discussion

TCFs are important downstream transcriptional effectors of MAPK signaling, which is normally correlated with the regulation of cell proliferation in mammalian cells (46). We and others have demonstrated that ELK4 is overexpressed in various cancers, including colorectal cancer and melanoma, and is required for cancer cell proliferation (19, 47). In this study, we established the important role of ELK4 in regulating mast cell proliferation but not mast cell development, which may impact disorders related to mast cell proliferation, such as mastocytosis. In addition to cell proliferation, ELK4 also plays a vital role in mast cell activation. Intriguingly, the effects of ELK4 on cytokine/chemokine expression and degranulation are opposites during mast cell activation. Although both *de novo* synthesis of cytokines/chemokines and degranulation are vital for mast cell function, the inflammatory response with the newly made cytokines/chemokines and degranulation are not always synchronous in response to different stimuli in mast cells. FcϵRI-mediated mast cell activation involves both cytokine/chemokine expression and degranulation, while MRGPRX2-mediated activation is mainly correlated with degranulation (48). Additionally, the response to FcϵRI crosslinking depends on the affinity of the antigens; high-affinity antigens lead to increased degranulation, and low-affinity antigens induce a more chemokine-based inflammatory response (49). Thus, it is worth exploring the mechanism of *Elk4* downregulation upon treatment with Compound 48/80 and IgE-DNP/HSA and the regulation and effect of ELK4 during mast cell activation under different stimuli in the future.

The MAPK pathway is also activated in mast cells in response to FcϵRI crosslinking (50–52). A recent study showed that inhibition of MEK1/2 resulted in increased cellular retention of β-hexosaminidase in IgE-DNP/HSA-stimulated BMMCs, which indicates decreased mast cell degranulation (53). Thus, although ELK4 is an important downstream transcriptional effector of MAPK, the MAPK pathway could promote mast cell degranulation, possibly through another downstream effector that needs to be explored in the future.

MITF is a core transcription factor for mast cell development and maintenance (54, 55). A recent study revealed that MITF is critical for *Hdc* gene expression through its binding to an upstream enhancer of *Hdc* in mast cells (38, 55). In this study, we identified MITF as a new interactor of ELK4; thus, we speculate that ELK4 might directly regulate *Hdc* and cytokine/chemokine transcription in mast cells by cooperating with MITF. Additionally, the TCF-SRF (serum response factor) transcriptional complex mediates the activation of various immediate-early genes, such as the AP1 transcription factor c-FOS, EGR1 (early growth response 1) and EGR2 (56). Transcriptional analysis of mast cells has revealed the vital role of AP1 and EGR1/2 in orchestrating the transcriptional inflammatory response for the *de novo* synthesis of cytokines and chemokines in activated mast cells (12, 57–59). The gene expression profile of the unstimulated *Elk4* KO BMMCs showed that c-Fos and EGR2/4 were both significantly downregulated by Elk4 deficiency (**Table S4H**). Thus, ELK4 could also indirectly regulate the inflammatory response of activated mast cells by activating AP1 and EGRs in mast cells, which also implies the potential role of SRF in mast cells.

The histone deacetylase SIRT6 is a recently identified negative regulator of mast cell degranulation that epigenetically inhibits target gene transcription (14). However, as a transcriptional corepressor, it was previously unknown which transcription factor recruits SIRT6 to regulate gene transcription in mast cells. Our study revealed that ELK4 exerts a comprehensive effect on the transcriptome in mast cells through both transcriptional activation and inhibition. Although the exact mechanism by which ELK4 suppresses mast cell degranulation is unknown, it is likely to involve a protein interaction with SIRT6. We hypothesize that ELK4 may recruit SIRT6 as a transcriptional corepressor to suppress gene transcription and inhibit degranulation. In addition to SIRT6, ELK4 is also responsible for the recruitment of SIRT7, which leads to deacetylation of H3K18Ac and oncogenic transformation in epithelial cancers (60). Thus, it would be promising to explore whether SIRT7 is involved in the transcriptional inhibitory effect of ELK4 in mast cells.

The limitations of this study mainly include the use of conventional *Elk4* KO mice and the lack of the genome-wide DNA binding profile of ELK4 in mast cells. Due to the lack of ChIP (chromatin immunoprecipitation)-grade ELK4 antibody and the technical difficulties of BMMC transfection and infection, we could not profile the DNA binding pattern of ELK4 in the mast cell system. Generating engineered mice expressing epitope-tagged endogenous ELK4 will provide a powerful tool to explore ELK4 binding profiles in different contexts, including mast cells (61). Additionally, mast cell-specific conditional *Elk4* KO mice will provide further *in vivo* evidence to support the vital role of ELK4 in mast cell biology. In summary, our study suggests that ELK4 interacts with MTIF and SIRT6 in mast cells and exerts opposite roles in the cytokine/chemokine production and degranulation during mast cell activation, which highlights the critical role of ELK4 in mast cell biology and anaphylaxis. Hence, combination of repressing ELK4 but promoting SIRT6 activity may point the way to developing new treatments for human diseases in which mast cells play a pathogenic role.

## Data availability statement

The raw RNA-seq data presented in this study are deposited in GEO repository, accession number: GSE236014.

## Ethics statement

The animal study was reviewed and approved by the Institutional Animal Care and Use Committee of Shanghai General Hospital affiliated with Shanghai Jiao Tong University School of Medicine. Written informed consent was obtained from the owners for the participation of their animals in this study.

## Author contributions

YH, C-YL, and LL conceived the idea of the study; C-YL and LL supervised the study; ZZ performed the bioinformatics analyses; WL and YL performed the animal experiment; YH performed all the *in vitro* assays with the help of YG; JW, XP, LHL, and JL contributed in the technical, material, or administrative support of the study. YH and C-YL wrote and edited the manuscript. All authors contributed to the article and approved the submitted version.

## Supplementary material

Supplementary Figure 1 Confirmation of BMMCs derived from *Elk4* WT, HZ and KO mice **(A)** Identification of mouse genotype by PCR and gel electrophoresis. **(B)** Identification of differentiated BMMCs after 8 weeks of differentiation. Cells were stained with CD117-FITC and FcϵRI-PECY7 and then analyzed by flow cytometry. **(C)** Granularity analysis of the differentiated BMMCs after 8 weeks of differentiation. Cells were stained with avidin-FITC and then analyzed by flow cytometry. **(D)** Toluidine blue staining images of BMMCs after 8 weeks of differentiation. **(E)** The ELK4 protein level in *Elk4* WT, HZ, and KO BMMCs assessed by western blot.

Supplementary Figure 2
*Elk4* deficiency does not affect other cell lineages, including T cells, B cells, macrophages and neutrophils **(A)** FACS analysis of cells from *Elk4* WT, HZ and KO mice. T cells and B cells were prepared from the spleen, and neutrophils (Neu) and macrophages (Mφs) were prepared from bone marrow.

Supplementary Figure 3 IL6, TNFα, CCL3 protein expression was decreased in *Elk4* KO BMMCs in response to FcϵRI-mediated activation **(A)**IL6, TNFα, CCL3 protein expression were assessed by flow cytometry analysis in Elk4 WT, HZ and KO BMMCs stained with IL6-eFluor 450, TNFα-APC and CCL3-PE. Elk4 WT, HZ and KO BMMCs were sensitized with anti-DNP-IgE (1 μg/ml) overnight and stimulated with DNP-HSA (100 ng/ml) for 1 hour before intracellular staining with IL6-eFluor 450, TNFα-APC and CCL3-PE. Mean Fluorescence intensity were analyzed statistically. Bar, mean; error bar, SD; n=3-5; *, p < 0.05; **, p<0.01. **(B)** IL6, TNFα, CCL3 protein level in supernatant of IgE-DNP/HSA stimulated BMMCs were assessed by ELISA assay. Elk4 WT, HZ and KO BMMCs were sensitized with anti-DNP-IgE (1 μg/ml) overnight and stimulated with DNP-HSA (100 ng/ml) for 1 hour. Bar, mean; error bar, SD; n=3; **, p<0.01.

Supplementary Figure 4
*Elk4* deficiency affects the release of histamine and beta-hexosaminidase from mast cells **(A, B)** The rate of histamine release (histamine in supernatant/total histamine in the cell) **(A)** and total content of histamine in BMMCs **(B)** were assessed. *Elk4* WT, HZ and KO BMMCs were sensitized with anti-DNP-IgE (1 μg/ml) overnight and stimulated with DNP-HSA (100 ng/ml) for 1 hour before quantification of histamine by ELISA. Bar, mean; error bar, SD; n=3; *, p < 0.05; **, p<0.01; ***, p<0.001. **(C, D)** Degranulation of *Elk4* WT, HZ and KO BMMCs **(C)** and PCMCs **(D)** was assessed by beta-hexosaminidase release assay. BMMCs and PCMCs were stimulated with Compound 48/80 (5 μg/ml) for 0.5 hours before analysis. One-way ANOVA with Dunnett’s multiple comparison test was used to assess statistical significance in this figure. Bar, mean; error bar, SD; n=3; **, p<0.01; ***, p<0.001.

Supplementary Figure 5 Cell cycle genes were altered in *Elk4*-deficient BMMCs **(A)**qPCR analysis of *E2f2*, *Cdk2*, *Cdk6*, *Ccna2*, and *Ccnb2* mRNA levels in unstimulated *Elk4* WT and KO BMMCs. Student’s t test was used to assess statistical significance. Bar, mean; error bar, SD; n=3; *, p < 0.05; **, p<0.01.

Supplementary Figure 6 An MITF inhibitor does not affect mast cell degranulation, while a SIRT6 inhibitor enhances mast cell degranulation **(A, B)** The rate of histamine release (histamine in supernatant/total histamine in the cell) in WT BMMCs treated with ML329 **(A)** and OSS-128167 **(B)** was assessed. One-way ANOVA with Dunnett’s multiple comparison test was used to assess statistical significance in this figure. Bar, mean; error bar, SD; n=3; **, p<0.01.
