# Supplementary figures and images for: ELK4 exerts opposite roles in cytokine/chemokine production and degranulation in activated mast cells
Source: Front Immunol. 2023 Jul 17;14:1171380. doi: 10.3389/fimmu.2023.1171380 (PMC10389778; doi:10.3389/fimmu.2023.1171380)

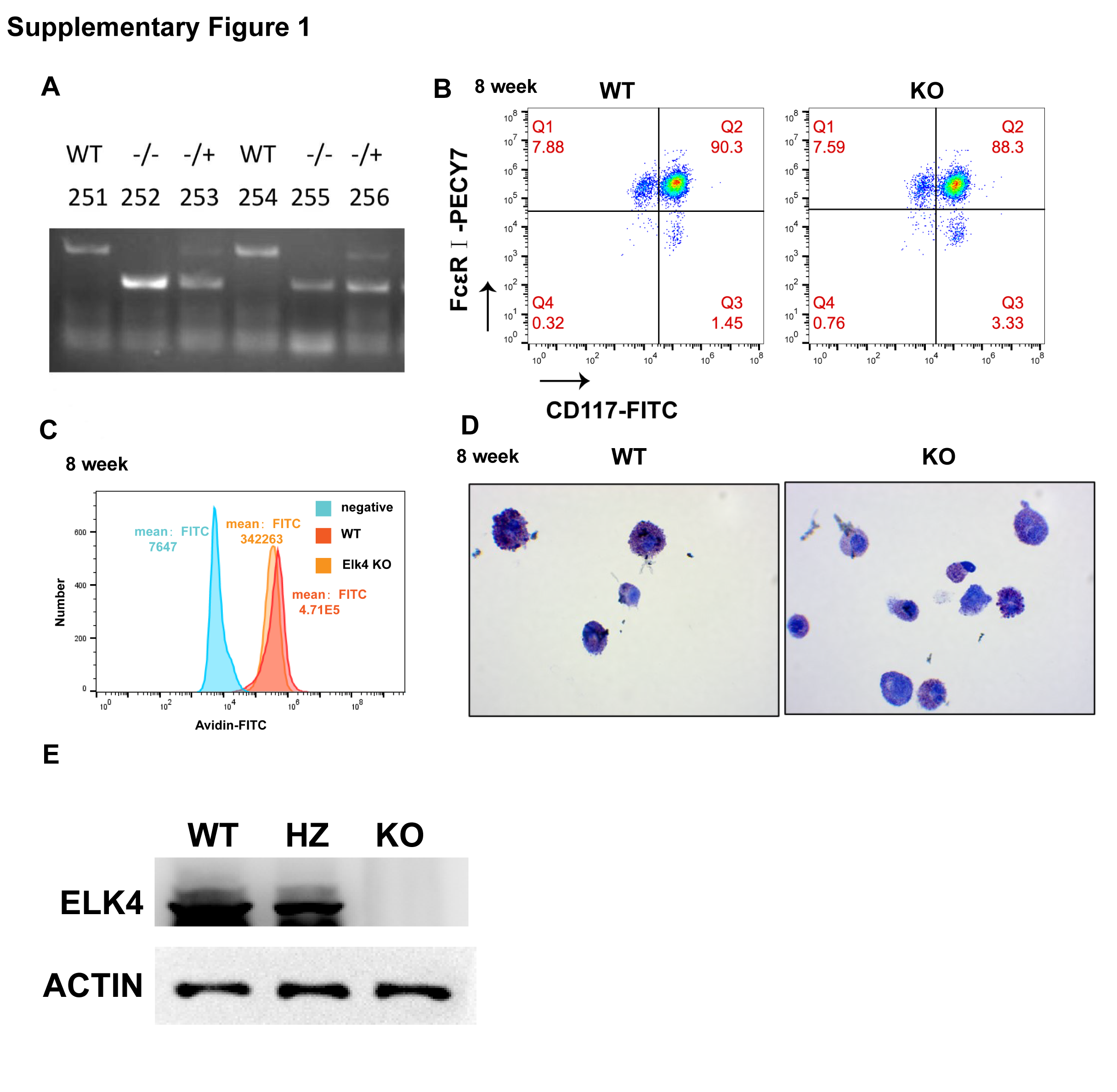

Supplement: Supplementary Figure 1 — Confirmation of BMMCs derived from Elk4 WT, HZ and KO mice (A) Identification of mouse genotype by PCR and gel electrophoresis. (B) Identification of differentiated BMMCs after 8 weeks of differentiation. Cells were stained with CD117-FITC and FcϵRI-PECY7 and then analyzed by flow cytometry. (C) Granularity analysis of the differentiated BMMCs after 8 weeks of differentiation. Cells were stained with avidin-FITC and then analyzed by flow cytometry. (D) Toluidine blue staining images of BMMCs after 8 weeks of differentiation. (E) The ELK4 protein level in Elk4 WT, HZ, and KO BMMCs assessed by western blot. [file Image_1.tif]

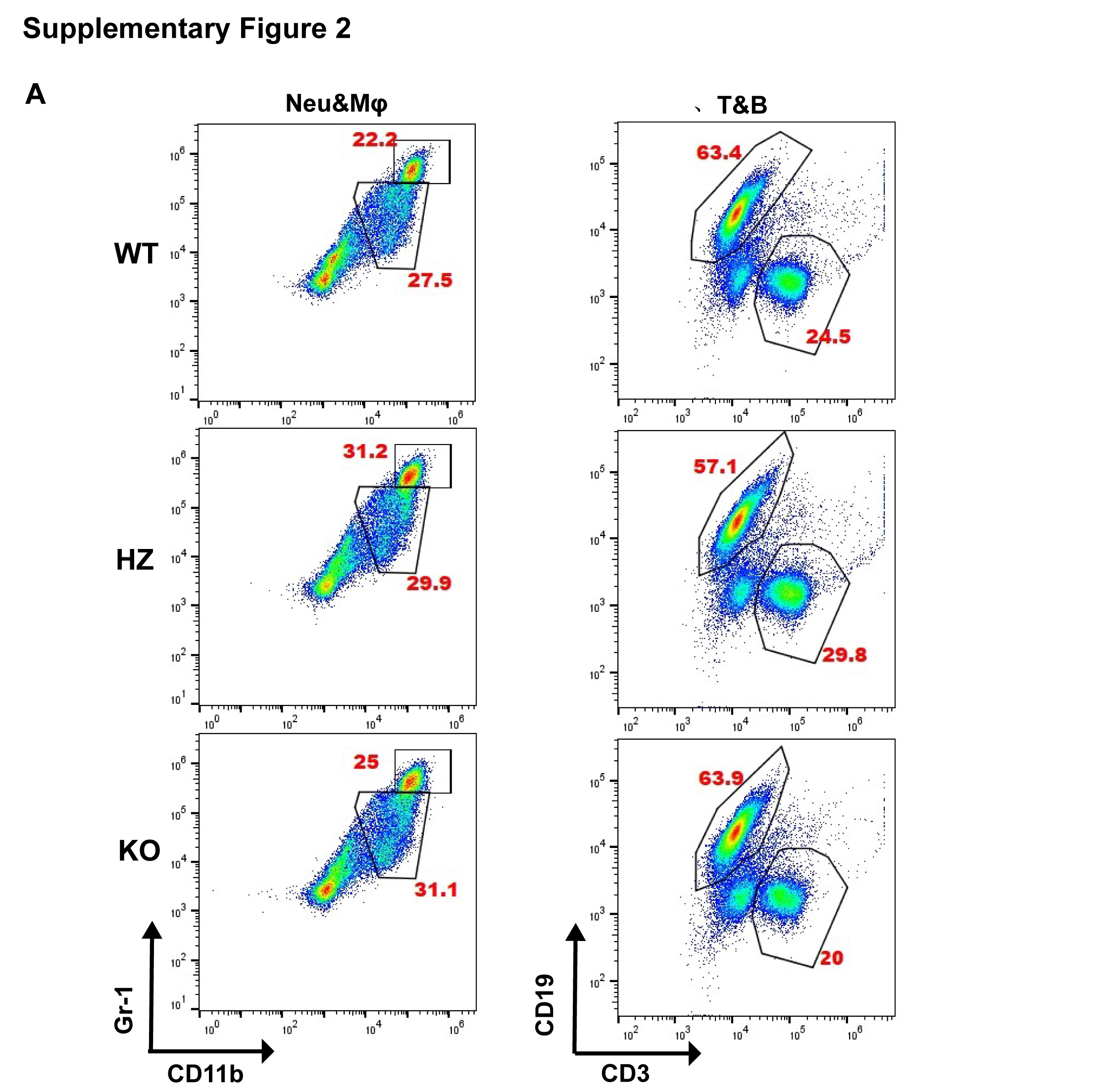

Supplement: Supplementary Figure 2 — Elk4 deficiency does not affect other cell lineages, including T cells, B cells, macrophages and neutrophils (A) FACS analysis of cells from Elk4 WT, HZ and KO mice. T cells and B cells were prepared from the spleen, and neutrophils (Neu) and macrophages (Mφs) were prepared from bone marrow. [file Image_2.tif]

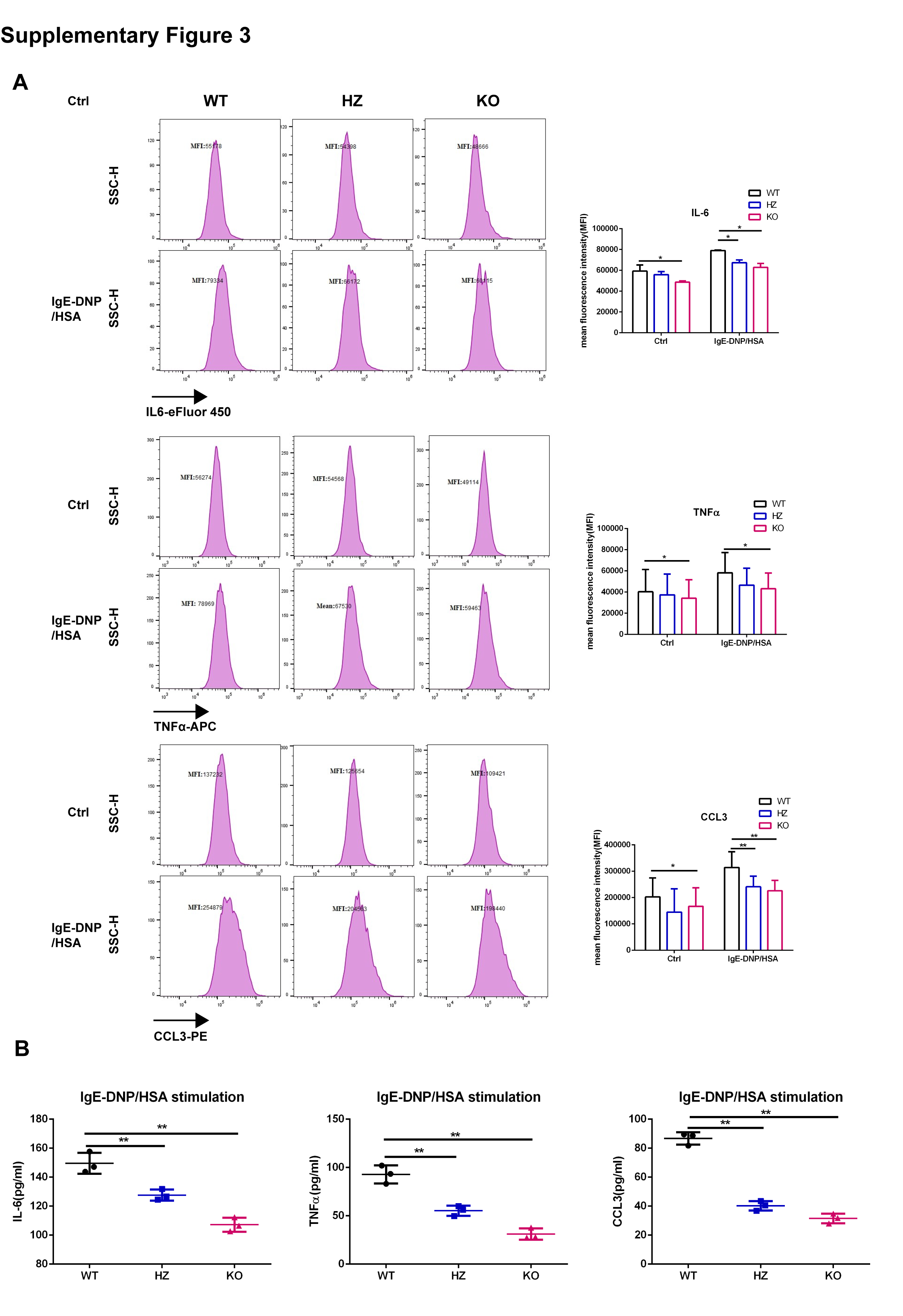

Supplement: Supplementary Figure 3 — IL6, TNFα, CCL3 protein expression was decreased in Elk4 KO BMMCs in response to FcϵRI-mediated activation (A)IL6, TNFα, CCL3 protein expression were assessed by flow cytometry analysis in Elk4 WT, HZ and KO BMMCs stained with IL6-eFluor 450, TNFα-APC and CCL3-PE. Elk4 WT, HZ and KO BMMCs were sensitized with anti-DNP-IgE (1 μg/ml) overnight and stimulated with DNP-HSA (100 ng/ml) for 1 hour before intracellular staining with IL6-eFluor 450, TNFα-APC and CCL3-PE. Mean Fluorescence intensity were analyzed statistically. Bar, mean; error bar, SD; n=3-5; *, p < 0.05; **, p<0.01. (B) IL6, TNFα, CCL3 protein level in supernatant of IgE-DNP/HSA stimulated BMMCs were assessed by ELISA assay. Elk4 WT, HZ and KO BMMCs were sensitized with anti-DNP-IgE (1 μg/ml) overnight and stimulated with DNP-HSA (100 ng/ml) for 1 hour. Bar, mean; error bar, SD; n=3; **, p<0.01. [file Image_3.tif]

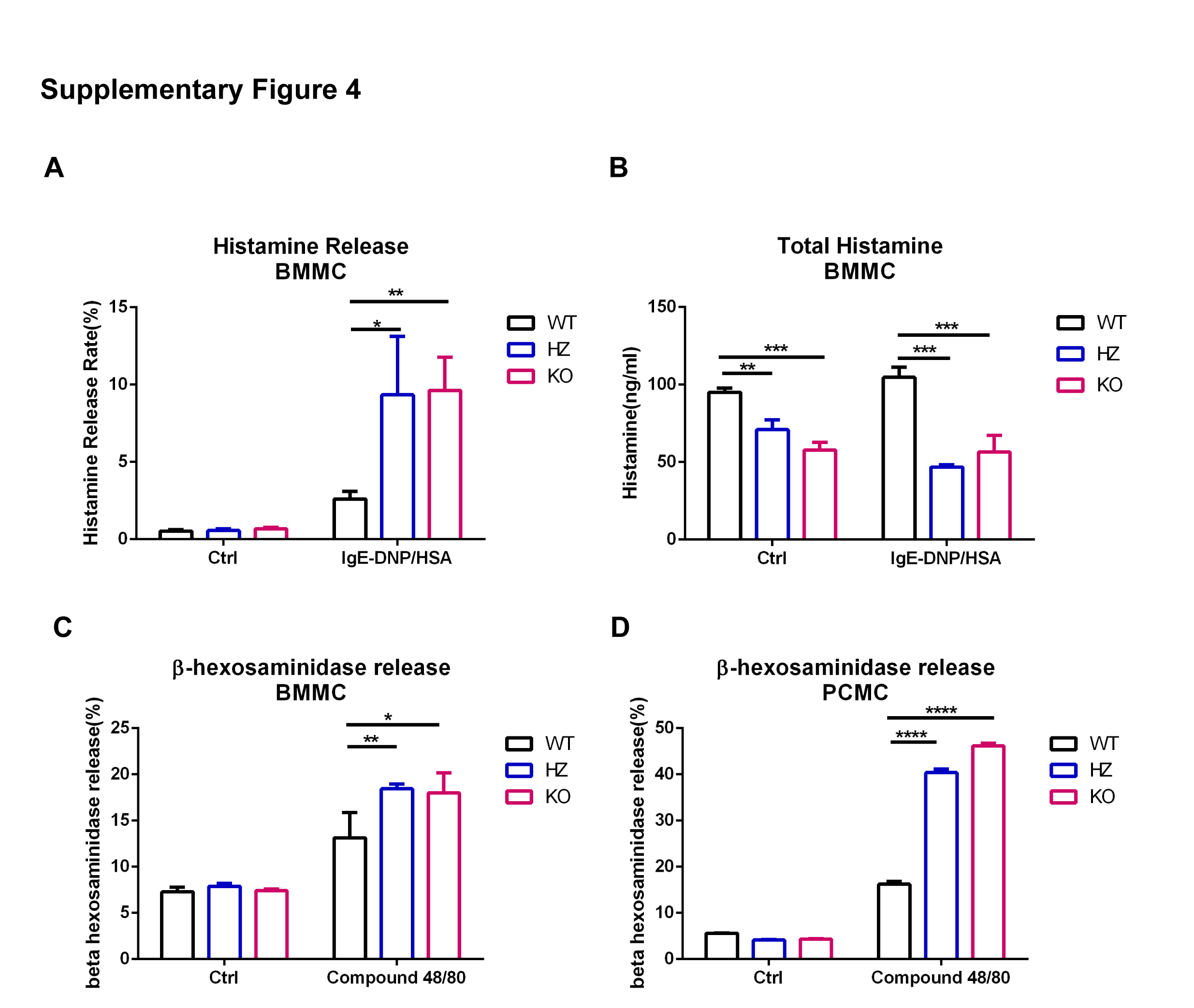

Supplement: Supplementary Figure 4 — Elk4 deficiency affects the release of histamine and beta-hexosaminidase from mast cells (A, B) The rate of histamine release (histamine in supernatant/total histamine in the cell) (A) and total content of histamine in BMMCs (B) were assessed. Elk4 WT, HZ and KO BMMCs were sensitized with anti-DNP-IgE (1 μg/ml) overnight and stimulated with DNP-HSA (100 ng/ml) for 1 hour before quantification of histamine by ELISA. Bar, mean; error bar, SD; n=3; *, p < 0.05; **, p<0.01; ***, p<0.001. (C, D) Degranulation of Elk4 WT, HZ and KO BMMCs (C) and PCMCs (D) was assessed by beta-hexosaminidase release assay. BMMCs and PCMCs were stimulated with Compound 48/80 (5 μg/ml) for 0.5 hours before analysis. One-way ANOVA with Dunnett’s multiple comparison test was used to assess statistical significance in this figure. Bar, mean; error bar, SD; n=3; **, p<0.01; ***, p<0.001. [file Image_4.tif]

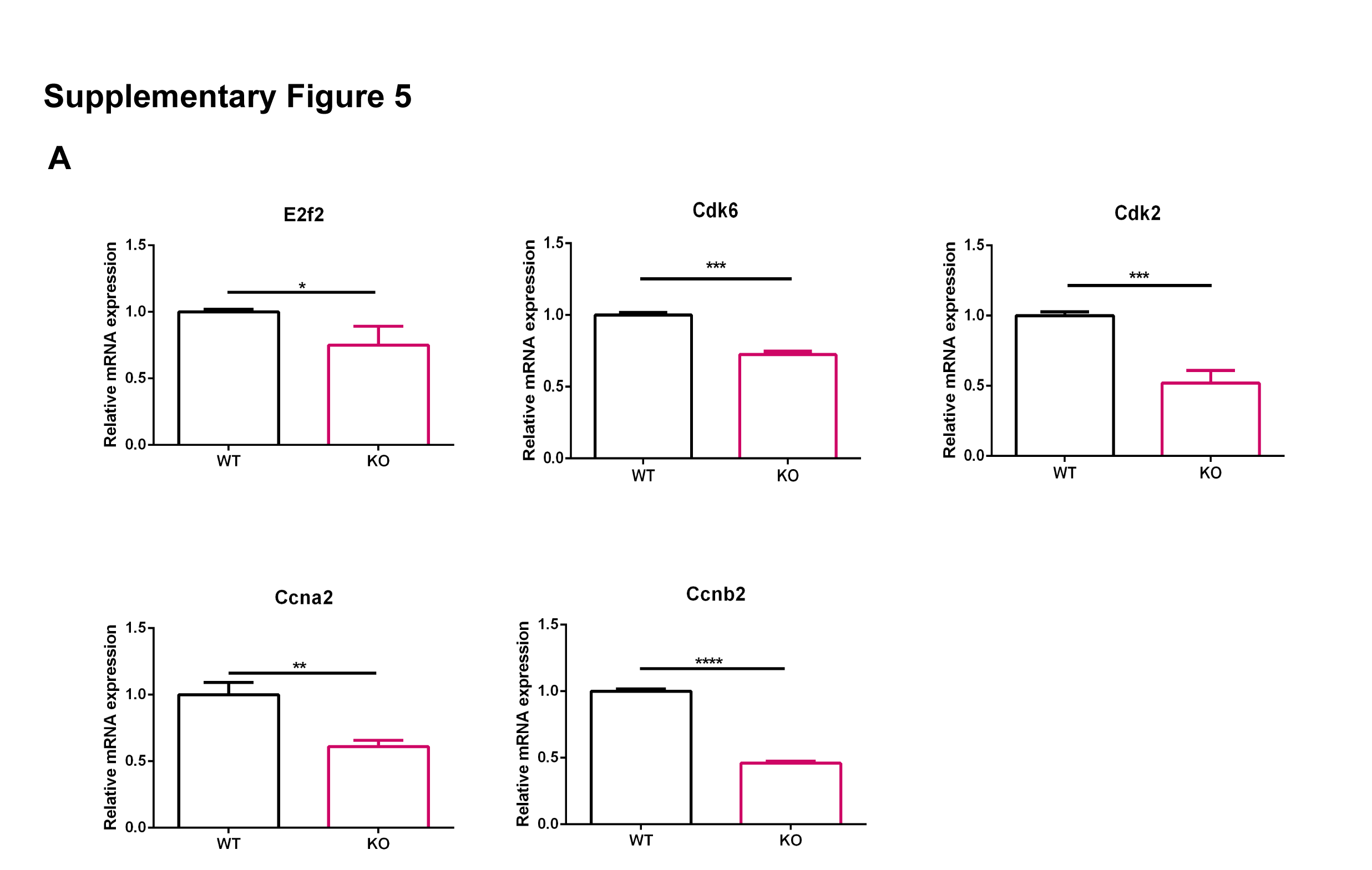

Supplement: Supplementary Figure 5 — Cell cycle genes were altered in Elk4-deficient BMMCs (A)qPCR analysis of E2f2, Cdk2, Cdk6, Ccna2, and Ccnb2 mRNA levels in unstimulated Elk4 WT and KO BMMCs. Student’s t test was used to assess statistical significance. Bar, mean; error bar, SD; n=3; *, p < 0.05; **, p<0.01. [file Image_5.tif]

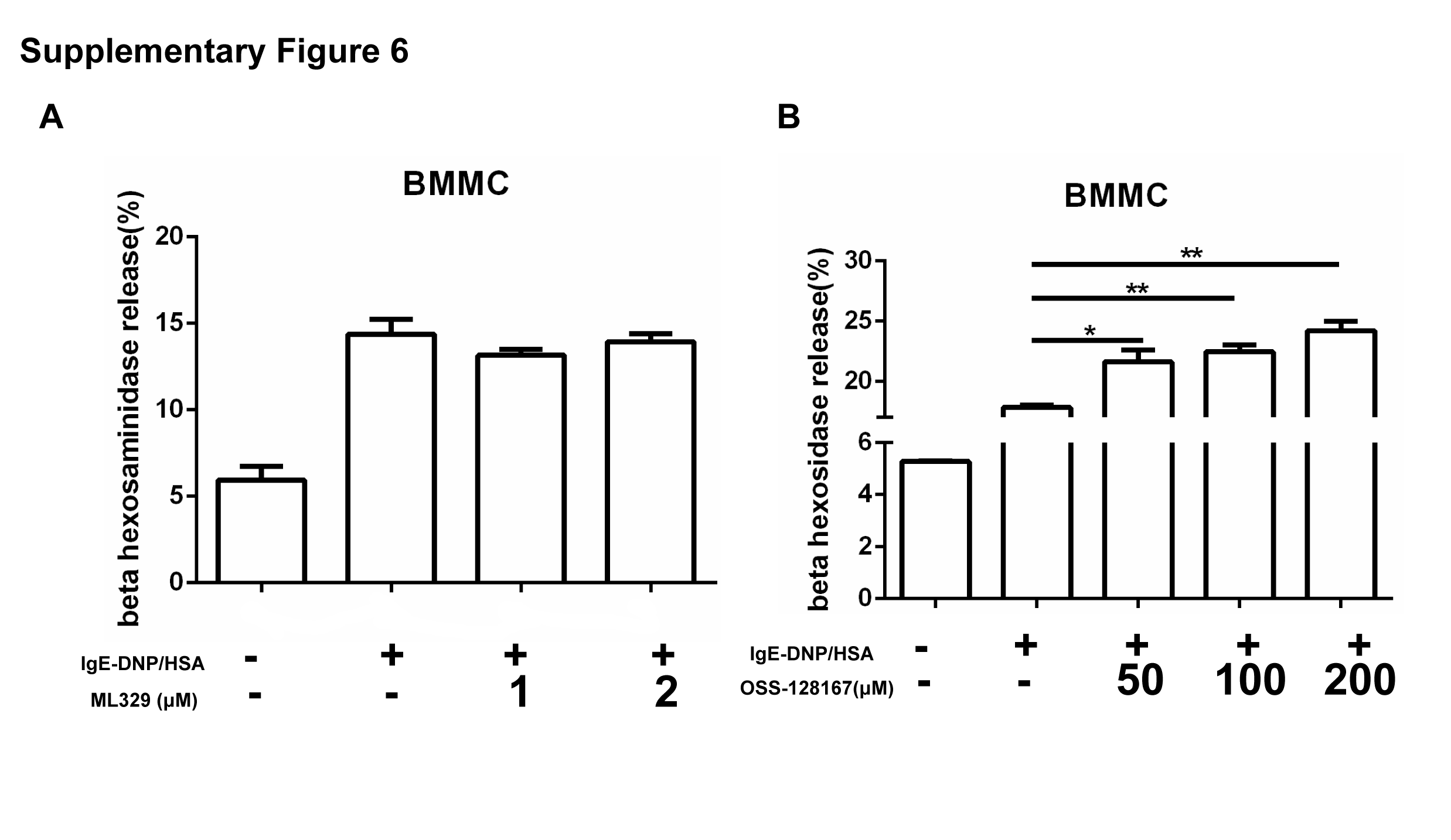

Supplement: Supplementary Figure 6 — An MITF inhibitor does not affect mast cell degranulation, while a SIRT6 inhibitor enhances mast cell degranulation (A, B) The rate of histamine release (histamine in supernatant/total histamine in the cell) in WT BMMCs treated with ML329 (A) and OSS-128167 (B) was assessed. One-way ANOVA with Dunnett’s multiple comparison test was used to assess statistical significance in this figure. Bar, mean; error bar, SD; n=3; **, p<0.01. [file Image_6.tif]
