# Supplementary material for: ELK4 exerts opposite roles in cytokine/chemokine production and degranulation in activated mast cells
Source: Front Immunol. 2023 Jul 17;14:1171380. doi: 10.3389/fimmu.2023.1171380 (PMC10389778; doi:10.3389/fimmu.2023.1171380)
Supplement: Supplementary file 7 [file Table_1.docx]

| genes | sequences |
| --- | --- |
| *Ccnb2* | F: GCCAAGAGCCATGTGACTAT  R: CAGAGCTGGTACTTTGGTGTTC |
| *Il13* | F: TGGTTTGCTGCCTATGCCCT  R: GCTACAGTGAGGTAGCAGAGTTA |
| *Il6* | F: GCCTTCTTGGGACTGATG  R: AGGTCTGTTGGGAGTGGTA |
| *Ccl3* | F: CAGCCAGGTGTCATTTTCCTGA  R: ATGCAGGTGGCAGGAATGTT |
| *Ccl4* | F: AAACCTAACCCCGAGCAACA  R: ATGCAGGTGGCAGGAATGTT |
| *Hdc* | F: TTCCAGCCTCCTCTGTCTGT  R: GGTATCCAGGCTGCACATTT |
| *E2f2* | F: ACGGCGCAACCTACAAAGAG  R: GTCTGCGTGTAAAGCGAAGTG |
| *Ccna2* | F: GCCTTCACCATTCATGTGGA  R: TTGCTCCGGGTAAAGAGACA |
| *Cdk6* | F: TCTCACAGAGTAGTGCATCGT  R: CGAGGTAAGGGCCATCTGAAAA |
| *Cdk2* | F: CCTGCTTATCAATGCAGAGGG  R: GTGCTGGGTACACACTAGGTG |
| *Hprt* | F: TCAGTCAACGGGGGACATAAA  R: GGGGCTGTACTGCTTAACCAG |

Table1 Primers used in RT-qPCR assay
